# Seldom heard voices: a meta-narrative systematic review of Aboriginal and Torres Strait Islander peoples healthcare experiences

**DOI:** 10.1186/s12939-020-01334-w

**Published:** 2020-12-14

**Authors:** Benjamin Jones, David Heslop, Reema Harrison

**Affiliations:** School of Public Health and Community Medicine, UNSW Sydney, Sydney, Australia

**Keywords:** Patient experience, Health services research, Aboriginal and Torres Strait Islander, Indigenous

## Abstract

**Background:**

It is well established that Aboriginal and Torres Strait Islander populations face considerable health inequities, exacerbated by poorer healthcare quality. Patient experience is recognised as a major contributing factor to healthcare quality and outcomes, therefore, enriched knowledge of the patient experiences of Aboriginal and Torres Strait Islander populations is critical to redress health inequities. This review synthesises evidence of the healthcare experiences amongst Aboriginal and Torres Strait Islander patients through a metanarrative synthesis of qualitative literature.

**Methods:**

A systematic search strategy was developed and applied to six electronic databases between January 2000 and July 2019. Titles and abstracts were screened before applying the inclusion criteria to full text articles. A meta-narrative synthesis was undertaken.

**Results:**

Fifty-four publications were identified from four research traditions; each with a unique conceptualisation of patient experience. Three themes emerged that demonstrate Aboriginal and Torres Strait Islander patient experiences are informed by 1) beliefs about wellbeing and healthcare provision, 2) their level of trust in the healthcare system, and 3) individual and community health system interactions. The findings highlight a range of aspects of patient experience that were important to participating Aboriginal and Torres Strait Islanders in the included studies but not captured currently in health system surveys.

**Conclusion:**

This review highlights the influence of beliefs about health and wellbeing on the patient experience amongst Aboriginal and Torres Strait Islander populations in the Australian health system. Patient experiences were informed by past experience and their trust in the health system. The different factors influencing patient experience and the gravity of their influence must be considered in current approaches to capturing patient experience data collection methods.

**Trial registration:**

PROSPERO (ID: CRD42019134765).

## Introduction

It is well established that Aboriginal and Torres Strait Islander populations face considerable health inequities [1–5]. One of the largest demonstrated inequity gaps in life expectancy in the world exists between Aboriginal and Torres Strait Islander and non-Indigenous Australians; with inter- and intra-ethnic disparity between those within these populations driven by factors such as socio-economic determinants [2]. Many reports published on this inequity conclude that improving patient experience will lead to better health outcomes [1, 3–5].

The term ‘patient experience’ describes the sum of all emotional and physical lived experiences that an individual has as they interact with the health system throughout the continuum of their care [6, 7]. These experiences include both the direct and non-direct, clinical and non-clinical interactions and are filtered through their world view [8, 9]. Individual’s expectations regarding the autonomy they have in their care and of their health outcomes also contribute to experience data [10].

Knowledge of patient (and carer) experience is widely considered as central to enhancing healthcare quality and safety [11–13]. Positive patient experiences are associated with higher healthcare quality, with these data making a key contribution to understanding and therefore mitigating potential safety risks [14]. Many health systems globally have therefore adopted national or organisational assessment of patient experience through large-scale survey programs to assess system performance [14–17].

An array of tools are used internationally to capture patient experience [18, 19]. Exploration of the nature of patient feedback collected through such tools indicates that current approaches are often resource intensive and limited in their application for improving healthcare. A key finding by Sheard et al. [20] was the dissonance between applications of patient experience data at macro- and micro-levels within healthcare services. Survey methods often reduce experience to a set of transactions upon which patients self-report. Substantial differences in beliefs around health and well-being apparent between and within Indigenous and non-Indigenous populations may therefore not be fully considered in current health system experience surveys in Australia [1, 3–5]. Increasingly, there is interest and recognition of the need to capture more nuanced and patient-centred data of experiences; that is to collect experience data about what the patient or carer identifies as an important part of their experience, specifically with a focus to what they would like to see improved [20–22].

In establishing the patient experience data currently omitted from health systems regarding the experiences of Aboriginal and Torres Strait Islanders, it is critical that we establish the evidence base to facilitate a bottom-up approach to understanding the features of healthcare that are important to this population. Our research therefore aimed to synthesise the substantial and disparate evidence base of patient and carer experience amongst Aboriginal and Torres Strait Islander peoples in the Australian health system to better understand experiences and the information missed by current mechanisms for experience data capture. We addressed the review question: what is known about the experiences of Aboriginal and Torres Strait Islander patients and carers in Australian healthcare settings?

## Methods

The Preferred Reporting Items for Systematic Reviews (PRISMA) was used to guide the reporting this study and the study protocol registered with PROSPERO (ID: CRD42019134765). An initial scoping review of the literature indicated that a significant volume of qualitative experience data were available across disparate literature. A meta-narrative approach was therefore adopted with the aim of developing a storyline of the Aboriginal and Torres Strait Islander patient experience research [23]. The review was conducted and reported using the Realist and Meta-narrative Evidence Syntheses: Evolving Standards (RAMSES) [24].

### Eligibility criteria

#### Inclusion criteria

Articles were included if they: were published between January 2000–June 2019, English language, included data from any sector of health service provision within the Australian health system, included a sample of Australian Aboriginal and Torres Strait Islander patients, any study design, and included Aboriginal & Torres Strait Islander patient-reported data regarding their experience defined as: *the sum of all emotional and physical lived experiences that an individual has as they interact with health services in Australia throughout the continuum of their care* [6, 25].

#### Exclusion criteria

Articles were excluded if they did not meet the inclusion criteria including literature capturing hypothetical experiences instead of personal reflections of experiences, and studies that focused on complaints or solely on accessing the health system rather than experiences of health services within the system.

### Study identification

Synonyms and relevant concepts were developed for these two major components in this review of *Australian Aboriginal and Torres Strait Islander* patients and *patient experience*. A search strategy (see Additional file 1) was developed and applied to the following electronic databases in July 2019: MEDLINE, PubMed, Informit, Scopus, Indigenous Health Info Net, and Web of Science. Results were merged using reference-management software (Endnote X9.2), duplicates were removed, and articles imported to Covidence.

### Study selection and data extraction

One reviewer (BJ) screened the titles and abstracts against the eligibility criteria. Full-text documents were obtained for all potentially relevant articles. The eligibility criteria was then independently applied to the articles by two reviewers (BJ, RH). A third reviewer conducted a face validity check on the final set of articles for inclusion or when there was disagreement on study eligibility (DH). The academic discipline backgrounds of the authors of this review were Aboriginal health, Medicine, Public Health, Health Services Research and Psychology. The following data were extracted from the included studies; author, date, method, analytic approach, sample size, healthcare service, objective, main findings, research tradition, academic discipline, state, and community.

### Assessment of study quality

All included studies were appraised against the ten criteria of the Critical Appraisal Skills Programme (CASP) Qualitative studies checklist, scored as ‘Yes’, ‘No’, or ‘Can’t tell’ and the results tabulated to obtain a sense of the strengths and limitations of the included work (see Additional file 2) [26]. The CASP tool was not used to exclude studies based on study quality.

### Data synthesis

Due to the qualitative nature of the included studies, data were synthesised using a meta-narrative approach in accordance with Greenhalgh (2005) [23]. A meta-narrative is the ‘storyline’ of the research findings that emerges from a specific research tradition. The meta-narrative was undertaken in six phases; planning, searching, mapping, appraisal, synthesis, and drawing conclusions. The research tradition and academic discipline of each study was explored through a series of discussions by the research team to determine the disciplines and research traditions contributing to the body of evidence. Summaries of how each research tradition had conceptualised patient experience were documented. Each study was also appraised individually by the research team through extended discussion between the team members (BJ, DH, RH) before framing the data through narrative synthesis.

## Results

### Results of the search

**Fig. 1:**
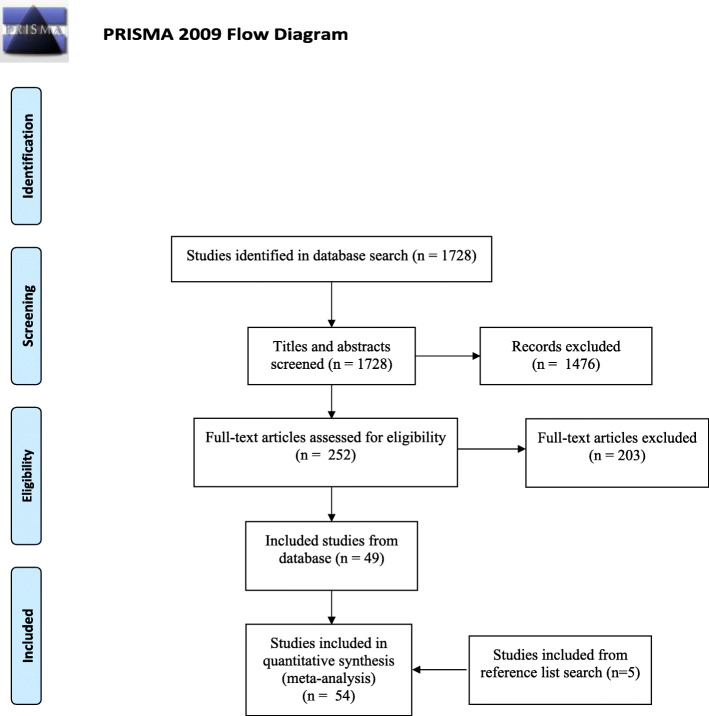
Study selection process

After duplicates were removed, 1728 papers were extracted from Endnote into Covidence. After title and abstract screening, 252 papers fulfilled the inclusion criteria and copies of full texts were obtained. Full text screening resulted in a total of 49 papers included in the review, with five additional papers included from reference list searches. A total of 54 papers were included in the review. Figure 1 presents the process.

### Excluded studies

The most common reasons for excluding papers were not focused on patient experience (119), focused on other international Indigenous populations such as the Maori, Canadian Aboriginal, Taiwanese, or First Nations American communities (54), reported staff member perceptions rather than patients and/or carers (22), referred to ideal hypothetical experiences (5), or focused on the experience of a research study not a health service (3).

### Characteristics of included studies

A total of 54 included studies yielded 50 unique data sets. Sample size ranged from 5 [27] to 282 [28], with a majority (42) having a sample size less than 50. Patient experience data were gathered via focus groups (11), interviews (42), open items in surveys (5), meta-synthesis of qualitative data (1), or prospective cohort study (1). Interviews were semi-structured (20), yarning style (7), unstructured interviews (6), or unspecified (11). Whilst not limited to one formal definition, yarning refers to a casual, friendly, but deep knowledge sharing style of conversation [29]. Settings included hospitals (20), Aboriginal community-controlled health organisations (10), primary health centres (5), discipline specific health services (8) or the broader health system (9). See Additional file 3 for a summary of included publications.

### Study quality

The CASP assessment of quality indicated that all of the included studies had many strengths in the way that the data were collected, ethical considerations, statements of findings, and the research value. Areas of weakness included a lack of clear statement of the aims of the research (12) which made it difficult to assess whether the research design and recruitment strategies were appropriate to address the aims of the research [30–41]. The majority of studies lacked consideration of the relationship between the researchers and research participants, with only seven studies commenting on this and potential resultant bias [42–48].

### Review findings

#### Research traditions

There were four academic research traditions that produced the data synthesised in this review that held particular conceptualisation of Aboriginal and Torres Strait Islander patient experience, resulting in four differing ‘storylines’ of the patient experience. Conceptualisations of patient experience were informed by authors’ academic disciplinary background and grounded by their research traditions; informed by assumptions, methodologies, and ways of framing findings [49]. The research traditions contributing to the meta-narrative were Medicine, Nursing, Public Health and Indigenous Public Health.

The papers written from a medical research tradition were characterised by their aim to evaluate a health service performance or compare Aboriginal and Torres Strait Islander experience to non-Indigenous experience, being positioned in a specific health discipline (e.g. cancer, renal, cardiac), and commonly used the term ‘patients’ to refer to research participants [28, 32, 42]. This research tradition resulted in a conceptualisation of patient experience that aligned with a Western biomedical model of health and healthcare and a more traditional patient-clinician dyad than other research traditions.

Papers founded within the nursing research tradition were characterised by; a patient advocacy tone with an impetus on the need for action, authors having closer relationships with the participants, an awareness of patient experience differences between patient groups, and referral to the research participants as consumers, clients, or participants [44, 50, 51]. Patient experience was therefore conceptualised more broadly to include the care environment and the support around an individual.

The Indigenous public health research tradition was characterised by; evidenced relationships with local community members in which a study was taking place, identified Aboriginal and Torres Strait Islander authors, were published in journals related to Indigenous health, and were informed by Indigenous research methods [52–54]. Indigenous research methods are research methodologies that are informed by, and value Indigenous cultural practices and customs [55]. This may include methods such as yarning style interviews, circle focus groups, or use of community leaders to identify participants and guide the researchers in navigating the community. This research tradition led to a conceptualisation of experience that was population-health based and embedded with an understanding of the Aboriginal and Torres Strait Islander model of health.

The general public health research tradition was distinguished from the Indigenous public health narrative by its anchoring to the Western model of health in terms of approach and understanding of health. For example, often these papers had an absence of stated Aboriginal researchers and substantial community consultation [56–58]. The conceptualisation here was centred on large cohorts and considering the population-wide implications of patient experience knowledge.

Three thematic areas emerged from the data: 1) *Beliefs around wellbeing and healthcare provision* which highlighted the strong influence of perspectives on health and wellbeing on patient experience; 2) *The impact of trust,* which describes the impact trust within systems and with providers on healthcare experiences and 3) *Health system interactions* that demonstrates how past positive and/or negative encounters with the health system influence patient experience*.*

#### Beliefs around wellbeing and healthcare provision

Included studies reflected the well-established notion that health is conceptualised by Aboriginal and Torres Strait Islanders differently to Western traditions [59]. For example, patients in nine of the studies alluded to the importance of being on Country^1^ when receiving healthcare to their overall patient experience [35, 47, 54, 60–65]. Being away from Country was seen as significant for many reasons including; missing important cultural events and ceremonies (funerals (sorry business), passing on of stories and customs (lore), and initiation ceremonies from childhood to adulthood), having competing priorities [38, 42, 56, 60, 61, 66], not feeling comfortable using resources on someone else’s Country [61], scared of dying off Country due to the spiritual connotations [61–63], and ultimately because of the importance of Country to wellbeing [35, 47, 54, 60–65]. These findings were pervasive in the data regardless of the research tradition and consistent across multiple settings and groups.

Feelings of intimidation were reported by Aboriginal and Torres Strait Islander people commonly which was often described as associated with lack of comfort with and/or health literacy in the Western model. Fear and feeling powerlessness, as well as uncertainty in which questions to ask healthcare providers, contributed to this feeling of intimidation [32, 35, 42, 46, 52, 56, 67–71]. This led to negative experiences including patient’s missing fundamental requirements of informed consent for tests and procedures [67] or a lack of awareness of reasons for admission [45]. Receiving health education material that was not culturally relevant [56] or the use of medical jargon by staff members also impacted negatively [56, 69, 72–74], effects which resolved or reversed when culturally relevant and competent interactions occurred [31, 33, 47, 51, 58, 68, 75–78]. Some patients also reported positive experiences in seeing Ngangkari - who are Aboriginal traditional healers [45].

The breadth of scope of patient experience, reflecting the Aboriginal model of holistic wellbeing, was recognised in many of the studies. Care was conceptualised in the broadest sense, with experience data regarding all aspects of the care process from the decision to seek care (often influenced by previous care experiences) to transport provision [34, 43, 46, 57, 58, 65, 73, 75, 78, 79], the continuity of care [42] and the number of follow-up processes that a service provides [30, 46, 56, 57, 73].

The impact of staff gender on experience was highlighted in eight studies [31, 48, 53, 56, 58, 66, 72, 74, 80]. The absence of gender specific hospital wards [66, 72, 74] and gender differences between patients and staff members (e.g. male patient with female doctor) were concerns raised on a number of occasions [31, 48, 53, 56, 58, 74, 80]. Family oriented administrative policies such as flexible visiting hours, no maximum number of visitors on wards [62, 65, 70, 80] and effective processes for family engagement with healthcare decision making were valued [32, 35, 42, 56, 62, 65, 70, 72, 78, 79, 81, 82].

Five studies reported that some Aboriginal and Torres Strait Islander people associated hospitals with death [30, 41, 42, 48, 72]. This view was more pronounced in rural and remote areas where tertiary hospitals and communication lines to communities may be dislocated, causing a vicious cycle of reducing confidence in healthcare, and delayed health seeking behaviours driving poorer outcomes. In contrast, despite differences in understanding healthcare, appreciation of professional, respectful staff and of confidentiality was found [31, 39, 42, 58]. Paradoxically, in some cases there was perceived concern around privacy of information in Aboriginal controlled-controlled health organisations due to the proximity of patient-staff relationships formed from being part of the same communities [31, 33, 81].

#### The importance of trust

Eighteen studies discussed the role of trust in systems in many Aboriginal and Torres Strait Islander’s patient experience and this was a significant and consistent finding across the traditions and their methodologies [34, 37, 42, 44, 46, 50, 52–54, 56, 58, 63, 70, 71, 76, 79, 81, 83]. Findings indicted that Aboriginal or Torres Strait Islander patients’ sense of trust in a health service is formed from a complex layering of experiences. At a basic level, the patient’s trust is informed by their experiences of current treatment in a particular health service, or more broadly the treatment of their current health complaint [27, 34, 44, 46, 50, 52, 53, 70, 79, 81, 83]. Many patients or carers also commented on the impact of historical traumas, particularly through the public health lens. These traumas, experienced previously when interacting with any part of the health system [37, 42, 56, 58, 63, 71], or with other non-health related institutions or harmful government policies, impact their patient experience. Community members’ experiences, and hear-say, also influenced many Aboriginal and Torres Strait Islander patient’s perceptions of what health services or providers could be trusted. These layers of mistrust manifest in several ways, from scepticism around the accuracy of staff explanations of diagnosis [52], to lack of trust in the transferring of their information and care plans between services [44], or a reluctance to share information with healthcare providers [40].

A number of positive elements of care enabled the development of trust for Aboriginal and Torres Strait Islanders. For example, 12 papers indicated that health professionals spending more time with a patient throughout the continuum of their care allowed for stronger and more trusting relationships to develop [31, 36, 42, 44, 50, 51, 53, 56, 69, 70, 75, 82]. Accordingly, where there was good continuity of care, this was also seen positively. The presence of Aboriginal and Torres Strait Islander staff was also valued [31, 32, 36, 38, 42, 45, 50, 53, 56, 81, 82], with one paper [50] concluding that “the Aboriginal Liaison Officer was perceived as shifting the power paradigm back in the direction of the patient and re-established their identity and place as clients within the system.”

#### Health system interactions

Thirty studies spanning the research traditions described the impact of interactions at all levels of the health system on experience [28, 32, 35, 37, 39, 40, 42, 44–46, 50–52, 54, 56, 57, 60, 61, 66, 67, 69, 71–75, 77, 80, 82–84], including with staff members, other patients, and with the physical space [6, 85]. Positive interactions with staff involved open, respectful, and culturally aware communication that included recognising the importance of not being limited by time restrictions [39, 45, 46, 60, 84]. Specifically, literature located within the nursing tradition identified a yarning style of conversation was highlighted as important [50, 51]. A yarning style was well-received by Aboriginal patients because the informal, relaxing nature of communicating as they would in the community allows people to feel more comfortable. Many papers also concluded that positive interactions were often with Aboriginal staff members due to their understanding of cultural dynamics [31, 33, 39, 50, 56, 58, 63, 70, 75, 80, 82].

Where health staff were perceived as lacking in openness, respect, used medical terminology jargon or did not adequately explain health-related concepts this led to poor experiences [32, 35, 40, 42–44, 52, 56, 73, 74]. Many patients reported feeling shame^2^ and judged by staff members, particularly in hospitals and mainstream health services [38, 40, 53, 57, 61, 71, 74] leading to feelings of disempowerment [66, 79]. Language barriers between the patients and the health staff/service were also prevalent in some areas of Australia where English is often not the first-language, particularly the Northern Territory, which created a barrier to good communication [37, 56, 60, 64, 67, 72, 74]. Literature acquired through a range of methodological perspectives consistently indicated the continued prevalence of racism within the health system [30, 36, 38, 42, 48, 56, 71, 74, 79, 82, 83].

Positive experiences of the physical environment were mentioned when a health service included symbols such as Aboriginal and Torres Strait Islander flags or artwork, or, had information flyers, posters etc. advertising local community events [36, 39, 63, 72]. Furthermore, the set-up of the waiting room in a circular, open configuration encouraged interactions with other patients [36]. Importantly, time spent in the waiting room in Aboriginal Medical Services provided an opportunity for solicited and unsolicited support from peers that was linked to satisfaction with the amount of time spent with the doctor [36].

Many of the negative experiences of physical space were reported in relation to the hospital environment [45, 70]. Across research traditions and through a variety of methodological approaches, patients reported discomfort being in the hospital environment, often due to negative past experiences [42, 56, 72], particularly when there were long waiting times [31, 32, 36, 38, 42, 45, 50, 53, 56, 81, 82]. Extended periods of waiting in some cases led to people feeling shame and helpless leading them to leave the service without being seen [36, 50, 81].

Respondents often valued the opportunity to interact with other patients at a service. Five studies referred directly to the positive impact that creating a community feeling to a health service had on the patient experience [30, 36, 57, 73, 81]. One study [27] found that seeing other community members at a service made them feel more comfortable. Another study [36] mentioned the benefit in being able to yarn about elements of their illness with other patients e.g. ‘how high are your sugars and what foods do you find keep them down?’ Evidence from Indigenous and broader public health traditions that look beyond the patient-clinician dyad highlighted the value of community events organised by health services to engage beyond the grounds of the health service and allow staff to connect with community and for patients to interact with one another [51, 58]. These elements contributed to some patients feeling pride in being a part of their local Aboriginal community health centre [75]. On the contrary, many studies reported the negative contribution that not knowing other patients in the health environment (particularly hospital environment) had on their comfort levels, and resulted in feelings of isolation [35, 44, 60] and loneliness [27, 45, 64, 66, 72].

## Discussion

This review provides synthesised knowledge of the extensive evidence-base regarding the Aboriginal and Torres Strait Islander patient experience in Australia. In this review, data from 54 publications provide, for the first time, a synthesised picture of Aboriginal and Torres Strait Islander experience across all health services ranging from hospitals, primary health centres, specialist care centres and Aboriginal health services.

We concluded that Aboriginal and Torres Strait Islander people have a nuanced experience of the health system which may impact their healthcare quality. This aligns with the ideas presented in a number of national reports on the Aboriginal and Torres Strait Islander patient journey [1, 3–5, 86]. It also aligns with the multi-dimensional concept of health and wellbeing pertaining to Indigenous health which speak to a person-centred approach [87]. The emphasis placed on what is important in a positive healthcare experience for Aboriginal and Torres Strait Islander people is focused to relational aspects of the care process rather than discrete transactional elements. One key difference was the emphasis placed on the development of trust with; a particular service, healthcare professionals, and the health system more broadly. Further, there was an evident strong desire to be understood holistically as a person within the health system, with social, mental, spiritual and community health needs considered in all interactions with the health system. Our findings demonstrate an imperative for an improved understanding of the needs of Aboriginal and Torres Strait Islander patients in the Australian health system and the need for a shift in methodology for capturing the breadth of patient experience data that is representative of the population served by a given health system.

Developing trust in the health system amongst Aboriginal and Torres Strait Islanders is fundamental towards creating a positive healthcare experience. Trust in the system and its many components has a two-fold benefit for experience. Trust firstly encourages people to engage with the health system i.e. to actively seek healthcare when required. This establishes the platform to have a positive experience. Secondly, trust encourages active participation of patients *within* the system, for example, sharing experiences and concerns in their entirety with health staff.

The qualitative literature explored in this review adds significant new knowledge to current quantitative state-wide healthcare experience data. To illustrate this, the New South Wales Bureau of Health Information’s Patient Perspectives Report – *Hospital Care for Aboriginal People* [88] importantly highlights that hospital care is largely positively rated by Aboriginal and Torres Strait Islander people. However, results differ in different areas, and in some specific aspects of care such as privacy and being given understandable answers, Aboriginal and Torres Strait Islander people were significantly less positive about their experiences than non-Indigenous people. However, this data cannot address the questions of; what made those experiences positive? Or, what specific factors made certain areas receive more positive scores than others? Or, why in specific aspects of care did Aboriginal and Torres Strait Islander people indicate their experiences were significantly less positive? These data are critical for healthcare quality improvement.

### Implications

There are a number of tangible opportunities to improve the experiences of Aboriginal and Torres Strait Islander people in the Australian healthcare setting. Recognising the unique challenges of the hospital environment for Aboriginal and Torres Strait Islander patients and carers in comparison to primary health centres and particularly Aboriginal Community-Controlled Health Services, there is an opportunity to target future resourcing to enhance patient and carer experiences within the hospital sector.

At an individual service level there are a number of culturally informed approaches that can be implemented. To facilitate this, there is a need for university health curriculums to better teach these approaches to ensure new entrants into the health workforce are culturally safe and better equipped to engage in positive interactions with their Aboriginal and Torres Strait Islander patients. These culturally informed approaches are well documented throughout the literature. As an example, one approach may be in relation to the physical space. The findings from this review suggest that many Aboriginal and Torres Strait Islander patients feel more welcomed in environments where their culture is featured [36, 39, 63, 72]. These features may be implemented through the design of waiting areas, the inclusion of outdoor spaces, or designs that allow for the opportunity to perform smoking ceremonies. The way in which the community element of Aboriginal culture is able to be embedded in the health service environment is another important consideration for health services. The notion of considering the service user, their background and the features of a welcoming environment from the consumer perspective has wider relevance.

A major implication of the review is a consideration for the optimal approach to measure the experience of Aboriginal and Torres Strait Islander people, which is relevant to many populations for whom current approaches may not be sufficient. The method by which experiential data is optimally elicited may not include the right questions to capture the full spectrum of Aboriginal and Torres Strait Islander peoples experience because they have not been developed with a knowledge of what is important to Aboriginal people. The inclusion of Aboriginal and Torres Strait Islander client focus groups or advisory councils may provide a mechanism to complement current survey methods in order to capture data regarding issues emerging as important in the present review.

In this review, it was evident that those studies utilising Indigenous research practices [55] when gathering experiential data were effective in facilitating patients and carers to speak freely and in-depth, resulting in new avenues of information about what matters in the Aboriginal and Torres Strait Islander patient experience [52–54]. Examples were the consultation of respected community members to identify participants or run sessions and the use of an informal, yarning style of interview. These practices allowed participants to feel more comfortable in sharing their experiences, enriching the data. Evidence of the need to consider a range of methods for capturing patient experience dependent on the purpose served indicates that diversity in the methods used is valuable to ensure the data is useable and useful [22].

Underpinning perceptions of good experience are patient and public perceptions about what constitutes good health and healthcare. Recognising differences in understandings of health and the provision of healthcare is therefore critical to enable service providers to meet patient needs but also for systems to assess and quantify positive experiences. The notion of being culturally knowledgeable is central to models of cultural competence for health professionals [89]. Similarly, being culturally knowledgeable at a health system level is necessary for the measurement of patient experience. This necessitates greater diversity in the approaches to measurement used and variation in content and focus across these measures. One example of variation in the framing and type of question would be to enable capture of experience amongst more collectivist population and cultural groups, which is lacking recognition in most patient experience surveys. The substantial influence of community and family member’s experiences on individuals may also be gathered by the patient and carer or ‘consumer’ being the focus of measurement tools.

In Australia, the capture of patient experience data amongst Aboriginal and Torres Strait Islanders necessitates discussion around who collects, owns, holds and grants access to the data. The author information for papers included in this review indicates that currently the data is largely collected by pockets of researchers working within research institutions who are interested in Aboriginal and Torres Strait Islander health who collaborate to explore the topic. For a national or health system level mechanism to be appropriate and towards developing trust, there may be a need to establish an Aboriginal and Torres Strait Islander governing body, possibly formed by the Aboriginal health peak organisations,^3^ that can set and regulate the standards for how patient and carer experience should be collected, where the data is held and who accesses this.

### Limitations

There were limitations to the process of the review and in the included studies. The omission of studies that did not ascribe to our definition of patient experience may have shaped the findings, in addition to limiting the review to only peer-reviewed published works. The accuracy of bibliographic databases may have impacted the selection of relevant material. The researcher’s perspectives, both academically and culturally, may have shaped the emerging concepts and resulting themes. The different disciplinary and cultural backgrounds of the team may have however enhanced our ability to explore the data from multiple viewpoints. The CASP quality assessment also revealed a number of areas of weakness in the body of the literature used for this review reported earlier. Another important limitation to acknowledge is that all Aboriginal and Torres Strait Islander communities differ and therefore the learnings from this review may not be relevant and applied in all communities. It is critical for health services to consult with the local Aboriginal and Torres Strait Islander community that their decisions may be impacting.

## Conclusion

This review demonstrates that the patient experience amongst Aboriginal and Torres Strait Islander populations in the Australian health system is substantially influenced by understandings of health and well-being, coupled with trust in healthcare systems and services. For Aboriginal and Torres Strait Islander patient experiences to be optimised, health systems must accurately gather these data and consider the range of factors that are significant for these population groups. Current approaches to the capture of patient experience data may not sufficiently enable this and should be examined with Aboriginal and Torres Strait Islander populations to co-create appropriate mechanisms for data collection and the action that follows.

## Supplementary Information

**Additional file 1.** Search strategy.

**Additional file 2.** Critical Appraisal Skills Programme (CASP) qualitative studies checklist.

**Additional file 3.** Summary of included publications.

## Data Availability

All data included in the review are publicly available research findings.

## Footnotes

1 Country is a term used by many Aboriginal and Torres Strait Islander Australians to describe the land of their family and ancestors. The relationship with the physical landscape and the associated feelings of connectedness when on Country are an important part of the Aboriginal and Torres Strait Islander culture.

2 ‘Shame’ is a specific term used by the Aboriginal community to reflect the deep feelings of embarrassment and uncomfortableness felt in certain circumstances.

3 The Aboriginal health peak organisations are the collection of national bodies that represent Aboriginal and Torres Strait Islander health services, allied health professionals, health workers, nurses, and doctors respectively.
