# Supplementary material for: Seldom heard voices: a meta-narrative systematic review of Aboriginal and Torres Strait Islander peoples healthcare experiences
Source: Int J Equity Health. 2020 Dec 14;19:222. doi: 10.1186/s12939-020-01334-w (PMC7734845; doi:10.1186/s12939-020-01334-w)
Supplement: Supplementary file 1 — Additional file 1. Search strategy. [file 12939_2020_1334_MOESM1_ESM.pdf]

Additional file 1

Search strategy

1. Oceanic Ancestry Group/
2. aborigin*.mp.
3. indigenous australia*.mp.
4. torres strait island*.mp.
5. koori.mp.
6. murri.mp.
7. tiwi.mp.
8. noongar.mp.
9. australian indigen*.mp.
10. australoid*.mp.
11. 1 or 2 or 3 or 4 or 5 or 6 or 7 or 8 or 9 or 10
12. patient experience*.mp.
13. patient satisfaction.mp. or exp Patient Satisfaction/
14. exp Patient-Centred Care/ or Patient-centred care.mp.
15. patient engagement.mp. or exp Patient Participation/
16. clinical interaction.mp.
17. exp Physician-Patient Relations/ or patient-clinician.mp.
18. clinian-patient.mp.
19. patient-doctor.mp.
20. doctor-patient.mp.
21. physician-patient.mp.
22. patient-physician.mp.
23. patient participation.mp.
24. patient reported experience*.mp.
25. patient reported experience measure*.mp.
26. 12 or 13 or 14 or 15 or 16 or 17 or 18 or 19 or 20 or 21 or 22 or 23 or 24 or 25
27. 11 and 26
