# Supplementary material for: Seldom heard voices: a meta-narrative systematic review of Aboriginal and Torres Strait Islander peoples healthcare experiences
Source: Int J Equity Health. 2020 Dec 14;19:222. doi: 10.1186/s12939-020-01334-w (PMC7734845; doi:10.1186/s12939-020-01334-w)
Supplement: Supplementary file 2 — Additional file 2. Critical Appraisal Skills Programme (CASP) qualitative studies checklist. [file 12939_2020_1334_MOESM2_ESM.pdf]

Additional file 2

Critical Appraisal Skills Programme (CASP) qualitative studies checklist

Name of study	Was there a clear statement of the aims of the research?	Is a qualitative methodology appropriate?	Was the research design appropriate to address the aims of the research?	Was the recruitment strategy appropriate to the aims of the research?	Was the data collected in a way that addressed the research issue?	Has the relationship between researcher and participants been adequately considered?	Have ethical issues been taken into consideration?	Was the data analysis sufficiently rigorous?	Is there a clear statement of findings?	How valuable is the research?
Anderson et al 2008 [1]	Yes	Yes	Yes	Yes	Yes	No	Yes	Yes	Yes	Valuable is stressing the importance of health education
Anderson et al 2012 [2]	No	Yes	Can't tell (More detailed account in other paper)	Can't tell (More detailed account in other paper)	Yes	No	Yes	No	Yes	Valuable in considering the various circumstances impacting compliance with treatment.
Artuso et al [3]	Yes	Yes	Yes	Yes	Yes	No	Yes	Yes	Yes	Valuable is highlighting health care utilisation factors.
Aspin et al [4]	Yes	Yes	Yes	Yes	Yes	No	Yes	Yes	Yes	Valuable in that the findings suggest that current non-Indigenous health care services are failing to meet the needs of Aboriginal and Torres Strait Islander people with chronic illness.
Baba, Brolan, Hil [5]	No (It states what the study provides	Yes	Can't tell (aims not clearly stated)	Can't tell (aims not clearly stated)	Yes	No	Yes	Yes	Yes	Valuable in calling for political action to improve services in a

	but not what it set out to do)									multi-sectoral approach e.g. housing, education, health.
Ban [6]	No (it states what was found but not aim)	Yes	Can't tell (aims not clearly stated)	Can't tell (aims not clearly stated)	Yes	No	No (no ethics approval)	No	Yes	Valuable is being the only entirely Torres Strait Islander focused paper.
Brener at al [7]	Yes	Yes (yes to some extent, interviews would have enriched the data over survey)et	Yes	Yes	No (interview may have been more appropriate to describe 'impact of culturally informed diagnosis')	No	Yes	Yes	Yes	Valuable in identifying the importance of the cultural appropriateness of diagnosis and the diagnosis process.
Brown [8]	Yes	Yes	Yes	Yes	Yes	Yes	Yes	Yes	Yes	Valuable is reviewing how to monitor Indigenous patients with ACS and determining the potential impact of system reform in the future.
Burnette & Kickett [9]	Yes	Yes	Yes	No (purposive sampling, only patient who spoke English were selected)	Yes	No	Yes	Yes	Yes	Valuable in highlighting need for more research in the space. Also recommends employment, cultural training, and provision of culturally safe care.
Campbell & Brown [10]	Yes	Yes	Yes	Yes	Yes	Yes	Yes	Yes	Yes	Valuable in identifying

										ACCHS are well placed to provide care to Indigenous women during pregnancy and postnatal period.
Canuto et al [11]	Yes	Yes	Yes	Yes	Yes	No	Yes	No	Yes	Valuable in recognising that Aboriginal men are interested in their health and are willing to share ideas on how it could be improved. Also, local strategies are good and future studies are needed on effectiveness of gender specific PHCs.
Chapman, Smith & Martin [12]	Yes	Yes	Yes	Yes	Yes	No	Yes	Yes	Yes	Valuable in assisting hospital to work in collaboration with Aboriginal community to develop and implement culturally appropriate care.
Cheng, Blum & Spain [13]	Yes	Yes (yes to some extent, interviews would have enriched the data over questionnaire)	Yes	Yes	Yes (yes to some extent, interviews would have further enriched the data)	No	Yes	Yes	Yes	Valuable in suggesting future studies could investigate targeted education resources.
Conway et al [14]	Yes	Yes	Yes	Yes	Yes	No	Yes	Yes	Yes	Valuable in recognising the positive

										benefits of the bus.
Corcoran et al [15]	Yes	Yes	Yes	Yes	Yes	Yes	Yes	Yes	Yes	Valuable in identifying the model of midwifery care that is important for Aboriginal patients.
Davies et al [16]	Yes	Yes	Yes	Yes	Yes	No	Yes	Yes	Yes	Valuable in recognising there is a desire for increased knowledge of Western model of health.
Dembinsky et al	Yes	Yes	Yes	Yes	Yes	No	Yes	Yes	Yes	Valuable in exploring palliative care patient experience
Devitt et al [17]	No (outlines what the study did but not what it originally aimed to do)	Yes	Can't tell (aims not clearly stated)	Can't tell (aims not clearly stated)	Yes	No	Yes	Yes	Yes	Valuable in recognising the need to better understand the cultural implications of organ transplants.
Einsiedel et al [18]	Yes	Yes	Yes	Yes	Yes	Yes	Yes	Yes	Yes	Valuable different approach and in call for further research.

Foley & Houston [19]	Yes	Yes	Yes	Yes	Yes	No	Yes	Yes	Yes	Valuable in highlighting the importance of providing good dietic care to Indigenous Australians.
Fredericks et al [20]	Yes	Yes	Yes	Yes	Yes	No	Can't tell	No	Yes	Valuable in developing and exploring a solution for improved life expectancy.
Freeman et al [21]	Yes	Yes	Yes	Yes	Yes	No	Yes	Yes	Yes	Valuable is exploring how to improve access.
Govil et al [22]	Yes	Yes	Yes	Yes	Yes	No	Yes	Yes	Yes	Valuable in its explanation of methodology and findings.
Green et al [23]	Yes	Yes	Yes	Yes	Yes	No	Yes	Yes	Yes	Valuable in its exploration of the challenges surrounding patient experience measurement for Indigenous peoples.
Harrington et al [24]	Yes	Yes	Yes	Yes	Yes	Yes	Yes	Yes	Yes	Valuable in understanding of different roles in the patient experiences.

Hepworth et al [25]	No (outlines what the study did but not what it originally aimed to do)	Yes	Can't tell (aims not clearly stated	Can't tell (aims not clearly stated	Yes	No	Yes	No	Yes	Valuable in reinforcing the benefit of culturally appropriate care.
Homer et al [26]	No (outlines what the study did but not what it originally aimed to do)	Yes	Can't tell (aims not clearly stated	Can't tell (aims not clearly stated	Yes	No	Yes	No	Yes	Valuable in exploring the important of community engagement and feedback in the design of a midwifery service.
Hughes et al [27]	No (outlines what the study did but not what it originally aimed to do)	Yes	Can't tell (aims not clearly stated	Can't tell (aims not clearly stated	Yes	No	No	No	Yes	Valuable in calling for a renal health reform.
Jan et al [28]	Yes	Yes	Yes	Yes	Yes	No	Yes	Yes	Yes	Valuable in highlighting the importance of qualitative data.
Jobling et al [29]	Yes	Yes	Yes	Yes	Yes	No	Yes	Yes	Yes	Valuable in understanding barriers and enablers.

Jowsey et al [30]	No (outlines what the study did but not what it originally aimed to do)	Yes	Can't tell (aims not clearly stated	Can't tell (aims not clearly stated	Yes	No	Yes	Yes	Yes	Valuable in understanding the experience of a people with chronic conditions.
Kelly et al [31]	Yes	Yes	Yes	Yes	Yes	No	Yes	No	Yes	Valuable in advocating for the increase of Indigenous midwives.
Lowell et al [32]	No (outlines what the study did but not what it originally aimed to do)	Yes	Can't tell (aims not clearly stated	Can't tell (aims not clearly stated	Yes	No	Can't tell	Yes	Yes	Valuable in recognising the importance of communication.
Mbuzi et al [33]	Yes	Yes	Yes	Yes	Yes	No	Yes	Yes	Yes	Valuable in recognising the unmet needs within the hospital system.
McGrath [34]	Yes	Yes	Yes	Yes	Yes	No	Yes	Yes	Yes	Valuable in recognising the importance for cultural context.
McMichael et al [35]	Yes	Yes	Yes	Yes	Yes	No	No	No	Yes	Valuable in allowing for a greater understanding of barriers to healthcare.
Meiklejohn et al [36]	Yes	Yes	Yes	Yes	Yes	No	Yes	Yes	Yes	Valuable in identifying the importance of strong relationships between people and

										their health providers.
Munro et al [37]	Yes	Yes	Yes	Yes	Yes	Yes	Yes	Yes	Yes	Valuable in recognising that experience is due to a multitude of factors.
Reilly et al [38]	Yes	Yes	Yes	Yes	Yes	Yes	Yes	Yes	Yes	Valuable in recognising that services can do more to facilitate positive interactions.
Rix et al 2014 [39]	Yes	Yes	Yes	Yes	Yes	No	Yes	Yes	Yes	Valuable in identifying the need for family-centred care.
Rix et al 2015 [40]	Yes	Yes	Yes	Yes	Yes	No	Yes	Yes	Yes	Valuable in highlighting that some Indigenous patients believe services are not flexible, optimally accessible, or family focused.
Shahid et al 2009 [41]	Yes	Yes	Yes	Yes	Yes	No	Yes	Yes	Yes	Valuable in advocating for the need of consumer directed care.
Shahid et al 2011 [42]	Yes	Yes	Yes	Yes	Yes	No	Yes	Yes	Yes	Valuable in exploring barriers faced.
Shahid et al 2016 [43]	No (outlines what the study did but not what	Yes	Can't tell (aims not clearly stated	Can't tell (aims not clearly stated	Yes	No	Yes	Yes	Yes	Valuable in identifying opportunities to improve cancer care and outcomes.

	it originally aimed to do)									
Smith et al [44]	No (outlines what the study did but not what it originally aimed to do)	Yes	Can't tell (aims not clearly stated	Can't tell (aims not clearly stated	Yes	No	Yes	Yes	Yes	Valuable in recognising need for culturally safe healthcare.
Strong et al [45]	No (outlines what the study did but not what it originally aimed to do)	Yes	Can't tell (aims not clearly stated	Can't tell (aims not clearly stated	Yes	No	Yes	Yes	Yes	Valuable in identifying the value of qualitative data in giving a voice to patients.
Tam et al [46]	Yes	Yes	Yes	Yes	Yes	No	Yes	Yes	Yes	Valuable in showing positive experience in cancer care.
Taylor et al [47]	No (outlines what the study did but not what it originally aimed to do)	Yes	Can't tell (aims not clearly stated	Can't tell (aims not clearly stated	Yes	No	Yes	Yes	Yes	Valuable in showing that AHWs significantly impact Aboriginal patients stay at hospital.

Thompson et al [48]	Yes	Yes	Yes	Yes	Yes	No	Yes	Yes	Yes	Valuable in recognising the importance of cultural structures and culturally safe care in the experience of Aboriginal and Torres Strait Islanders.
Treloar et al [49]	Yes	Yes	Yes	Yes	Yes	No	Yes	Yes	Yes	Valuable in exploring if Indigenous patients feel included in cancer care.
Webster et al [50]	Yes	Yes	Yes	Yes	Yes	No	Yes	Yes	Yes	Valuable in looking into health education and the perspective of the patient.
Worrall-Carter et al [51]	Yes	Yes	Yes	Yes	Yes	No	Yes	Yes	Yes	Valuable is understanding the disorientating effect for some Indigenous people of being taken off country for care.
Wotherspoon et al [52]	Yes	Yes (yes to some extent, interviews would have enriched the data over survey)et	Yes	Yes	Yes (yes to some extent, interviews would have further enriched the data)	No	Yes	Yes	Yes	Valuable in exploring strategies to improve health outcomes.

References

1. Anderson K, Devitt J, Cunningham J, et al. "All they said was my kidneys were dead": Indigenous Australian patients' understanding of their chronic kidney disease. *Med J Aust* 2008;189(9):499-503.
2. Anderson K, Cunningham J, Devitt J, et al. "Looking back to my family": Indigenous Australian patients' experience of hemodialysis. *BMC Nephrol* 2012;13 doi: Artn 114
10.1186/1471-2369-13-114
3. Artuso S, Cargo M, Brown A, et al. Factors influencing health care utilisation among Aboriginal cardiac patients in central Australia: a qualitative study. *BMC Health Serv Res* 2013;13 doi: Artn 83
10.1186/1472-6963-13-83
4. Aspin C, Brown N, Jowsey T, et al. Strategic approaches to enhanced health service delivery for Aboriginal and Torres Strait Islander people with chronic illness: a qualitative study. *BMC Health Serv Res* 2012;12 doi: Artn 143
10.1186/1472-6963-12-143
5. Baba JT, Brolan CE, Hill PS. Aboriginal medical services cure more than illness: a qualitative study of how Indigenous services address the health impacts of discrimination in Brisbane communities. *International Journal for Equity in Health* 2014;13 doi: Artn 56
10.1186/1475-9276-13-56
6. Ban P. Access and attitudes to health care of Torres Strait Islanders living in mainland Australia. *Australian Journal of Primary Health* 2004;10(2):29-35.
7. Brener L, Wilson H, Jackson LC, et al. Experiences of diagnosis, care and treatment among Aboriginal people living with hepatitis C. *Aust N Z J Public Health* 2016;40:59-64. doi: 10.1111/1753-6405.12402
8. Brown A. Acute Coronary Syndromes in Indigenous Australians: Opportunities for Improving Outcomes Across the Continuum of Care. *Heart, Lung and Circulation* 2010;19(5-6):325-36. doi: 10.1016/j.hlc.2010.02.011
9. Burnette L, Kickett M. 'You are just a puppet': Australian Aboriginal people's experience of disempowerment when undergoing treatment for end-stage renal disease. *Renal Society of Australasia Journal* 2009;5(3):113-18.
10. Campbell S, Brown S. Maternity care with the Women's Business Service at the Mildura Aboriginal Health Service. *Aust N Z J Public Health* 2004;28(4):376-82. doi: DOI 10.1111/j.1467-842X.2004.tb00447.x
11. Canuto K, Wittert G, Harfield S, et al. "I feel more comfortable speaking to a male": Aboriginal and Torres Strait Islander men's discourse on utilizing primary health care services. *International Journal for Equity in Health* 2018;17(185) doi: ARTN 185
10.1186/s12939-018-0902-1
12. Chapman R, Smith T, Martin C. Qualitative exploration of the perceived barriers and enablers to Aboriginal and Torres Strait Islander people accessing healthcare through one Victorian Emergency Department. *Contemp Nurse* 2014;48(1):48-58. doi: Doi 10.1080/10376178.2014.11081926
13. Cheng WYC, Blum P, Spain B. Barriers to effective perioperative communication in indigenous australians: an audit of progress since 1996. *Anaesth Intensive Care* 2004;32(4):542-47. doi: Doi 10.1177/0310057x0403200412
14. Conway J, Lawn S, Crail S, et al. Indigenous patient experiences of returning to country: a qualitative evaluation on the Country Health SA Dialysis bus. *BMC Health Serv Res* 2018;18 doi: ARTN 1010
10.1186/s12913-018-3849-4
15. Corcoran PM, Catling C, Homer CSE. Models of midwifery care for Indigenous women and babies: A meta-synthesis. *Women and Birth* 2017;30(1):77-86. doi: 10.1016/j.wombi.2016.08.003

16. Davies J, Bukulatjpi S, Sharma S, et al. "Only your blood can tell the story" - a qualitative research study using semi-structured interviews to explore the hepatitis B related knowledge, perceptions and experiences of remote dwelling Indigenous Australians and their health care providers in northern Australia. *BMC Public Health* 2014;14 doi: Artn 1233
10.1186/1471-2458-14-1233
17. Devitt J, Anderson K, Cunningham J, et al. Difficult conversations: Australian Indigenous patients' views on kidney transplantation. *BMC Nephrol* 2017;18 doi: ARTN 310
10.1186/s12882-017-0726-z
18. Einsiedel LJ, van Iersel E, Macnamara R, et al. Self-discharge by adult Aboriginal patients at Alice Springs Hospital, Central Australia: insights from a prospective cohort study. *Aust Health Rev* 2013;37(2):239-45. doi: 10.1071/Ah11087
19. Foley W, Houston A. Closing the gap by increasing access to clinical dietetic services for urban Aboriginal and Torres Strait Islander people. *Nutr Diet* 2014;71(4):216-22. doi: 10.1111/1747-0080.12090
20. Fredericks B, Kinnear S, Daniels C. Exploring knowledge, experiences and perceptions of chronic health conditions. *Aboriginal and Islander Health Worker Journal* 2016;40:17-18.
21. Freeman T, Edwards T, Baum F, et al. Cultural respect strategies in Australian Aboriginal primary health care services: beyond education and training of practitioners. *Aust N Z J Public Health* 2014;38(4):355-61. doi: 10.1111/1753-6405.12231
22. Govil D, Lin I, Dodd T, et al. Identifying culturally appropriate strategies for coronary heart disease secondary prevention in a regional Aboriginal Medical Service. *Australian Journal of Primary Health* 2014;20(3):266-72. doi: 10.1071/Py12117
23. Green M, Anderson K, Griffiths K, et al. Understanding Indigenous Australians' experiences of cancer care: stakeholders' views on what to measure and how to measure it. *BMC Health Serv Res* 2018;18(1):982.
24. Harrington Z, Thomas DP, Currie BJ, et al. Challenging perceptions of non-compliance with rheumatic fever prophylaxis in a remote Aboriginal community. *Med J Aust* 2006;184(10):514-17.
25. Hepworth J, Askew D, Foley W, et al. How an urban Aboriginal and Torres Strait Islander primary health care service improved access to mental health care. *International Journal for Equity in Health* 2015;14 doi: ARTN 51
10.1186/s12939-015-0183-x
26. Homer CSE, Foureur MJ, Allende T, et al. 'It's more than just having a baby' women's experiences of a maternity service for Australian Aboriginal and Torres Strait Islander families. *Midwifery* 2012;28(4):509-15. doi: 10.1016/j.midw.2011.06.004
27. Hughes JT, Dembski L, Kerrigan V, et al. Gathering Perspectives - Finding Solutions for Chronic and End Stage Kidney Disease. *Nephrology* 2018;23:5-13. doi: 10.1111/nep.13233
28. Jan S, Conaty S, Hecker R, et al. An holistic economic evaluation of an Aboriginal community-controlled midwifery programme in Western Sydney. *Journal Health Services Research Policy* 2004;9(1):14-21. doi: 10.1258/135581904322716067 [published Online First: 2004/03/10]
29. Jobling K, Lau P, Kerr D, et al. Bundap Marram Durn Durn: Engagement with Aboriginal women experiencing comorbid chronic physical and mental health conditions. *Aust N Z J Public Health* 2016;40:30-35. doi: 10.1111/1753-6405.12382
30. Jowsey T, Yen L, Ward N, et al. It hinges on the door: Time, spaces and identity in Australian Aboriginal Health Services. *Health Sociology Review* 2012;21(2):196-207. doi: DOI 10.5172/hesr.2012.21.2.196
31. Kelly J, West R, Gamble J, et al. 'She knows how we feel': Australian Aboriginal and Torres Strait Islander childbearing women's experience of Continuity of Care with an

Australian Aboriginal and Torres Strait Islander midwifery student. *Women and Birth* 2014;27(3):157-62. doi: 10.1016/j.wombi.2014.06.002

32. Lowell A, Maypilama E, Yikaniwuy S, et al. "Hiding the story": Indigenous consumer concerns about communication related to chronic disease in one remote region of Australia. *Int J Speech Lang Pathol* 2012;14(3):200-08. doi: 10.3109/17549507.2012.663791
33. Mbuzi V, Fulbrook P, Jessup M. Indigenous cardiac patients' and relatives' experiences of hospitalisation: A narrative inquiry. *J Clin Nurs* 2017;26(23-24):5052-64. doi: 10.1111/jocn.14005
34. McGrath P. Exploring Aboriginal peoples' experience of relocation for treatment during end-of-life care. *Int J Palliat Nurs* 2006;12(3):102-08. doi: 10.12968/ijpn.2006.12.3.20692
35. McMichael C, Kirk M, Manderson L, et al. Indigenous women's perceptions of breast cancer diagnosis and treatment in Queensland. *Australia and New Zealand Journal of Public Health* 2000;24(5):515-9. [published Online First: 2000/12/08]
36. Meiklejohn JA, Garvey G, Bailie R, et al. Follow-up cancer care: perspectives of Aboriginal and Torres Strait Islander cancer survivors. *Support Care Cancer* 2017;25(5):1597-605. doi: 10.1007/s00520-016-3563-x
37. Munro A, Allan J, Shakeshaft A, et al. "I just feel comfortable out here, there's something about the place": staff and client perceptions of a remote Australian Aboriginal drug and alcohol rehabilitation service. *Subst Abuse Treat Prev Policy* 2017;12 doi: ARTN 49

10.1186/s13011-017-0135-0

38. Reilly R, Micklem J, Yerrell P, et al. Aboriginal experiences of cancer and care coordination: Lessons from the Cancer Data and Aboriginal Disparities (CanDAD) narratives. *Health Expect* 2018;21(5):927-36. doi: 10.1111/hex.12687
39. Rix EF, Barclay L, Stirling J, et al. 'Beats the alternative but it messes up your life': Aboriginal people's experience of haemodialysis in rural Australia. *British Medical Journal Open* 2014;4(9) doi: ARTN e005945

10.1136/bmjopen-2014-005945

40. Rix EF, Barclay L, Stirling J, et al. The perspectives of Aboriginal patients and their health care providers on improving the quality of hemodialysis services: A qualitative study. *Hemodialysis International* 2015;19(1):80-89. doi: 10.1111/hdi.12201
41. Shahid S, Finn L, Bessarab D, et al. Understanding, beliefs and perspectives of Aboriginal people in Western Australia about cancer and its impact on access to cancer services. *BMC Health Serv Res* 2009;9 doi: Artn 132

10.1186/1472-6963-9-132

42. Shahid S, Finn L, Bessarab D, et al. 'Nowhere to room ... nobody told them': logistical and cultural impediments to Aboriginal peoples' participation in cancer treatment. *Aust Health Rev* 2011;35(2):235-41. doi: 10.1071/Ah09835
43. Shahid S, Teng THK, Bessarab D, et al. Factors contributing to delayed diagnosis of cancer among Aboriginal people in Australia: a qualitative study. *British Medical Journal Open* 2016;6(6) doi: ARTN e010909

10.1136/bmjopen-2015-010909

44. Smith K, Fatima Y, Knight S. Are primary healthcare services culturally appropriate for Aboriginal people? Findings from a remote community. *Australian Journal of Primary Health* 2017;23(3):236-42. doi: 10.1071/Py16110
45. Strong J, Nielsen M, Williams M, et al. Quiet about pain: Experiences of Aboriginal people in two rural communities. *Aust J Rural Health* 2015;23(3):181-84. doi: 10.1111/ajr.12185

46. Tam L, Garvey G, Meiklejohn J, et al. Exploring Positive Survivorship Experiences of Indigenous Australian Cancer Patients. *Int J Environ Res Public Health* 2018;15(1) doi: ARTN 135
10.3390/ijerph15010135
47. Taylor KP, Thompson SC, Smith JS, et al. Exploring the impact of an Aboriginal Health Worker on hospitalised Aboriginal experiences: lessons from cardiology. *Aust Health Rev* 2009;33(4):549-57. doi: Doi 10.1071/Ah090549
48. Thompson SC, Shahid S, Bessarab D, et al. Not just bricks and mortar: planning hospital cancer services for Aboriginal people. *BMC Res Notes* 2011;4:62. doi: 10.1186/1756-0500-4-62 [published Online First: 2011/03/16]
49. Treloar C, Gray R, Brener L, et al. "I can't do this, it's too much": building social inclusion in cancer diagnosis and treatment experiences of Aboriginal people, their carers and health workers. *International Journal of Public Health* 2014;59(2):373-9.
50. Webster E, Johnson C, Kemp B, et al. Theory that explains an Aboriginal perspective of learning to understand and manage diabetes. *Aust N Z J Public Health* 2017;41(1):27-31. doi: 10.1111/1753-6405.12605
51. Worrall-Carter L, Daws K, Rahman MA, et al. Exploring Aboriginal patients' experiences of cardiac care at a major metropolitan hospital in Melbourne. *Aust Health Rev* 2016;40(6):696-704. doi: 10.1071/Ah15175
52. Wotherspoon C, Williams CM. Exploring the experiences of Aboriginal and Torres Strait Islander patients admitted to a metropolitan health service. *Aust Health Rev* 2019;43(2):217-23. doi: 10.1071/Ah17096
