# Supplementary material for: Seldom heard voices: a meta-narrative systematic review of Aboriginal and Torres Strait Islander peoples healthcare experiences
Source: Int J Equity Health. 2020 Dec 14;19:222. doi: 10.1186/s12939-020-01334-w (PMC7734845; doi:10.1186/s12939-020-01334-w)
Supplement: Supplementary file 3 — Additional file 3. Summary of included publications. [file 12939_2020_1334_MOESM3_ESM.pdf]

Additional file 3

Summary of included publications

Author	Date	Method	Analysis	Sample	Healthcare service	Objective	• Main Findings	State	Mob/community
Anderson et al (26)	2008	Semi-structured interviews	Thematic analysis	146 Indigenous and 95 Non-Indigenous patients.	Hospital renal wards and dialysis centres	To explore the views of Indigenous Australians with ESKD about barriers to healthcare.	• Uncertainty about cause of illness distressing<br>• Do not feel adequately informed<br>• Less confident in knowledge of Western medicine<br>• Some think staff deliberately withhold information<br>• Scepticism and mistrust in staff explanations	-	-
Anderson et al (25)	2012	Semi-structured interviews	Thematic analysis	146 Indigenous and 95 Non-Indigenous patients.	Hospital renal wards and dialysis centres	To understand how dialysis impacts Indigenous patients.	• Unaware of severity of illness therefore diagnosis and treatment is a shock<br>• Being away from Country, community and missing ceremonies is hard<br>• Physical discomfort compounded by distress alienation<br>• Isolated<br>• Confused and sad<br>• Uncomfortable and uncertain so restricted engagement<br>• Clear communication without hiding facts is valued<br>• Language barriers	-	-

Artuso et al (49)	2013	Unstructured interviews then focus groups and semi-structures interviews.	Thematic analysis and a mixed coding method combine deductively and inductively derived codes.	7 Aboriginal cardiac patients, 15 Aboriginal non-cardiac community member and 12 health care providers and community researchers.	Any/Not specified	To identify factors that influence health care utilisation in Aboriginal cardiac patients.	Individual level:<br>• Language barriers<br>• Uninformed<br>• Lack of understanding around need for follow-up<br>• Negative associations with hospitals<br>• Mistrust due to past negative experiences<br>• Racism<br>• Lack of cultural awareness<br>• Time needed to build trust<br>• Competing priorities<br>• Fear<br>• Avoiding relationships with AHW<br><br>Interpersonal level:<br>• Communication between staff and patient's family<br>• Support for family attendance<br><br>Hospital system level:<br>• Miscommunication due to medical jargon<br>• Not enough focus on follow up care<br>• Waiting times<br>• Health education Westernised<br>• Need for gender specific doctors<br>• Importance of AHW<br>• Perception of being told what to do as an Aboriginal vs discussing options as non-Aboriginal.<br>• Western biomedical model of health conflicts	NT	Alice Springs
Aspin et al (58)	2012	Semi-structured interviews	Thematic analysis	16 Aboriginal or Torres Strait Islander people with chronic illnesses and 3 family members/carers.	Any/Not specified	To identify the facilitators of and barriers to healthcare among Aboriginal and Torres Strait Islander people living with chronic illnesses	• Intimidated due to previous experiences<br>• Intimidation intensified by community member's experiences<br>• Feeling judged and assumptions made<br>• All of this contributes too not following up<br>• Appreciate community and social atmosphere of AMS<br>• Long-term relationship with staff beneficial	NSW ACT	-

Baba, Brolan, Hil (28)	2014	Focus groups and semi-structured interview	Thematic analysis	22 Aboriginal or Torres Strait Islander patients of Aboriginal Community Controlled Health Services in Queensland	Any/Not specified	To provide a picture of the perspectives of Aboriginal and Torres Strait Islanders in accessing as AMS and identify self-perceived health determinants.	• Mainstream services do not accommodate for Indigenous concepts of health<br>• Follow-up needed<br>• Not enough emphasis on continuity of care leading to confusion about what to do next<br>• Not knowing good questions to ask<br>• Negative intergenerational experiences<br>• Understanding that hospital is a place to die<br>• Racist remarks<br>• More comfortable at AMS<br>• Importance of connection and story telling	QLD	Brisbane
Ban (29)	2004	Focus groups and individual interviews	Thematic analysis	92 Torres Strait Islanders. 16 from Alice Springs, 17 from Broome, 17 from Adelaide, 20 from Sydney and 22 from Cairns	Community controlled health services, Private medical clinics, Hospital outpatient services, Community health centres	To examine the attitudes towards and access to health services of Torres Strait Islanders living in mainland Australia	Community controlled health services:<br>• Cultural sensitivity<br>• Supporting Indigenous organisations<br>• Time given to patients<br>• Familiarity with service<br>• Social nature<br>• Concerns about confidentiality, lack of professionalism and long waiting times.<br>• Private medical clinics:<br>• Convenient<br>• Professional<br>• Can choose gender of doctor<br>• More about treatment less social occasion<br>Hospital outpatient service:<br>• Confidentiality strong<br>• Professional<br>• Importance of AHW<br>• Afraid of hospitals<br>• Only attend when really need to<br>• Negative of not being with community<br>• Community health centres:<br>• Confidentiality<br>• Adequate time with doctors<br>• Indigenous specific programs positive<br>• Professional	-	Torres Strait Islanders

Brener at al (60)	2015	Phone survey	Pearson product moment correlation then Spearman rank correlation and SPSS V21.	203 Indigenous patients (117 male, 84 female, 1 transgender)	Any service involved in HIV care	To describe HCV care and treatment among Indigenous Australians with HCV.	• 10% had never received information of HCV<br>• About the same percentage chose a GP as an AMS to receive HCV care<br>• Discrimination related to Aboriginality with a score on Group-Based Medical Mistrust Scale of 3.2	NSW	-
Brown (39)	2010	Open-ended interviews	Thematic analysis	110 Indigenous patients (or their next-of-kin for participants who passed away after their ACS)	Hospitals	To explore barriers and enablers to care for Indigenous patients who experience an acute cardiac event	• Delayed presentation due to competing priorities<br>• Mistrust due to past negative experiences<br>• Limited understanding of Western medicine leading to fear of consequences<br>• Community member's negative experiences compound<br>• Disrespectful treatment at hospitals<br>• Also many experience of 'good care', with professional, respectful staff.<br>• Waiting times<br>• Poor communication contributing to fear<br>• Feeling invisible especially when anxious<br>• Experiences of racism<br>• Important that family is very involved in all aspects of care<br>• Not enough time to form relationship in hospital<br>• Fear of death at hospital due to community members going the 'that big hospital down south to die'<br>• Misconception of being fixed after leaving hospital<br>• Not sure what to do post discharge	NT	Alice Springs and Darwin

Burnette & Kickett (50)	2009	Narrated ethnographic approach with conversational style interviews.	Thematic analysis	6 Aboriginal patients of the Royal Perth Hospital satellite dialysis and home therapies unit	Royal Perth Hospital satellite dialysis and home therapies unit	To explore the experience of treatment of end-stage-renal disease for Aboriginal patients.	• Overall experiences related to disempowerment<br>• Felt misunderstood by staff<br>• Tension of treatment vs community obligations e.g. Sorry business or cultural Lore<br>• Often unable to relate to staff<br>• Different understanding of importance of dialysis treatment<br>• Concerns around hospital alleviated if other Aboriginal people in ward<br>• Gender issues e.g. mixed gender wards<br>• Appreciated camaraderie with some health professionals	WA	Perth hospital dialysis units (3/6 were Noongar)
Campbell & Brown (40)	2004	Interviews modified from a questionnaire	Coding framework for open ended questions and quantitative used SPSS	25 Aboriginal women	Aboriginal health service	To assess and contrast experiences of women attending an AHS with those of rural women attending a public maternity service	• Felt informed.<br>• Overall felt that they had received good care. Specifically appreciated, home visits, sensitivity, reassurance, respect, education, transport availability.<br>• Felt information in hospital was sometimes contradictory or confusing	NT	Alice Springs
Canuto et al (48)	2018	Semi-structured on-on-one interview	Thematic analysis	19 Indigenous patients (10 Indigenous patients from Far north Queensland and 9 patients South Australia)	Primary health services including Aboriginal health services	To explore perceived motivators, barriers and enablers for primary care services utilisation amongst Aboriginal and Torres Strait Islander men.	• Feeling shame<br>• Waiting times too long<br>• Prefer AMS or if not most convenient service<br>• At AMS doctors ask about life not just illness (holistic approach to health)<br>• More time allows relationship formation and more trust<br>• Gender specific staff preferred	QLD, SA	Urban except 2 in FNQ

Chapman, Smith & Martin (54)	2014	Semi-structured focus group yarns	Thematic analysis	16 Aboriginal patients (12 women and 4 men)	Emergency department	To identify perceived barriers and enablers to accessing healthcare at the Emergency Department for the Aboriginal and Torres Strait Islander population.	• Waiting times stressful due to feeling vulnerable whilst waiting<br>• Lack of transparency<br>• Limited identification of Aboriginal patients due to staff's preconceived ideas of what Aboriginal patients may look like<br>• Feeling that other groups have certain things e.g. religious groups having access to religious contacts within the hospital<br>• Trust in AHW - "the ALO was perceived as shifting the power paradigm back in the direction of the patient and re-established their identity and place as clients within the system"<br>• Appreciated effort of staff<br>• Appreciated when dignity was maintained<br>• Taking time to sit and yarn appreciated	VIC	-
Cheng, Blum & Spain (24)	2004	Questionnaire	-	282 Aboriginal patients	Hospital	To explore barriers to perioperative communication between Aboriginal patients and anaesthetists.	• Communication difficulty<br>• Agree with doctor even if not understanding<br>• Interpreter important if English isn't sufficient	NT	Darwin

Conway et al (68)	2018	In-depth yarning style interviews	Thematic analysis	25 (15 patients (10 female and 5 male) and 10 staff)	Country Health SA Dialysis Bus	To evaluate the South Australian Mobile Dialysis Truck program, its impact on wellbeing and the facilitators and barriers to using the service.	• Impact of moving away from Country, the land and people<br>• Can't teach younger generations off Country<br>• Missing cultural events for treatment saddening<br>• Dying off Country (spiritual connotations),<br>• Shame for taking up local community's treatment space<br>• Bus is positive in keeping connection with Country<br>• Bus also facilitated better relationships because people felt at home and not dislocated<br>• Flexibility and cultural awareness are key	SA	Rural, remote SA, Alice Springs
Corcoran et al (41)	2017	Meta-synthesis of qualitative studies	Thematic analysis	9 studies (6 from Australia and 3 from Canada) (Australian papers two did not record participant numbers. Of the four that did they had 320 participants in total, 83 women who used the services, 18 midwives, two Aboriginal Health Workers, five Aboriginal Maternal Infant Care workers, one community member, 194 health staff and 17 external stakeholders)	Midwifery clinic	To review the experience s of Aboriginal women with midwifery models of care.	• Continuity of care positive<br>• Time for developing a relationship<br>• Appreciate not having to repeat info<br>• Sometimes feeling lost in mainstream system<br>• Distrust in information sharing and communication between centres can be limited<br>• Isolated in hospital<br>• Spoken down to in hospital by staff<br>• Feeling special in specific Indigenous programs	-	-

Davies et al (69)	2014	Semi-structured interviews	Deductive and inductive thematic analysis	32 interviews with patients (11), community members (9) and health professionals 12). 24 were Indigenous, 17 used Yolgnu Matha interpreter	Health clinic in Arnhem land and liver clinic in Darwin Hospital.	To explore the experiences and thoughts of Indigenous Australians who live remotely of Hepatitis B infection.	• Western concepts foreign and difficult to relate to<br>• Asked to do tests but not sure why<br>• Language barriers<br>• Communication is critical	NT	Arnhem Land/Yolgnu and Darwin
Dembinsky et al (79)	2014	Informal, yarning interviews	Thematic analysis	10 Aboriginal patients, 10 carers, 5 staff	Palliative care services	To examine the lived experiences of breast cancer for Yamatji women.	• Scared of dying off Country because of spirit connotations. Sense of closure from this and allow the proper ceremonies.<br>• Rooms too small for family visits.<br>• Lack of privacy in hospital.<br>• Not allowed to perform smoking ceremonies.<br>• Confusion over which services on offer and who to contact.	WA	Yamatji

Devitt et al (27)	2017	Semi-structured interviews	Thematic analysis	146 indigenous patients	Networks of hospital transplant units and dialysis treatment centres. 26 research sites.	To explore Indigenous ESKD patients' views on transplantation and treatment more broadly.	• Of 127 patients explicitly asked, 59 felt not well informed and did not understand treatments<br>• "Clinicians may well have spoken to some patients, but little had actually been communicated."<br>• ¼ didn't know waiting list status<br>• Complexity of content<br>• Difficulty understanding specialist communication manner<br>• Over assertiveness of health professionals,<br>• Feeling that staff use power to restrict information sharing (We would like to be spoken to clearly in an understandable way by doctors – …by doctors who like Anangu (Aboriginal people), by understanding doctors who talk - they're good – a lot of other doctors can't talk with us… their talk is hard [to understand].). Take the time.<br>• Access to interpreter important,<br>• Often don't discuss health with other patients<br>• Seeking family consensus on treatment option<br>• Lack of knowledge contributes to fear<br>• Cultural conflicts of Western medicine I.e. transplants,	-	-

Einsiedel et al (42)	2013	Prospective cohort study with interviews conducted in primary language by AHW's.	Statistical analysis	202 Indigenous inpatients	Alice Springs Hospital	To explore the cultural and environmental contexts in which self-discharge occurs in Central Australia.	• 6.4% complained of poor communication<br>• 2 patients were not satisfied with their medical care<br>• Understanding of reason was not consistent with physician in 73.4% of cases<br>• 29 were unable to give any reason for being in hospital<br>• 23.3% were concerned by hospitalisation<br>• 35.% were worried about elements of the hospital infrastructure (lifts, asphalt, being on second floor)<br>• 46.7% were concerned by idea of tertiary referral, and 55.6% about referral to Adelaide or Darwin<br>• 23.6% had seen Ngangkari<br>• 300 Aboriginal adults self-discharged during 489 admissions between July 2006 and August 2007<br>• 19.8% of interviewees self-discharged during study period<br>• In preceding decade 49.1% self-discharged<br>• Numerous reasons were given including loneliness, taken by family, payday, attending court, football, felt well, waiting too long, and the frequency of nursing observations<br>• Some were concerned with aspects of hospitals physical environment<br>• Many saw Ngangkari<br>• Most felt good about medical care received and only a few complained about communication.	NT	Alice Springs

Foley & Houston (55)	2013	In-depth interviews	Thematic analysis	13 Indigenous patients with diabetes	Inala Indigenous Health Service	To explore if changes to a dietetic service improved attendance and referrals and to learn what is important in dietetic service delivery	• Staff presence at community events<br>• Cultural understanding<br>• Communication styles<br>• More time in consultations<br>• Don't feel judged<br>• Culturally accessible resources<br>• Appreciate practical education<br>• Using appropriate terms like Aunty and Uncle<br>• Yarning style<br>• Everyday language	QLD	Brisbane
Fredericks et al (46)	2016	Focus groups, yarning interviews		14 Aboriginal people with chronic health conditions	Any	To explore the patient experience of Aboriginal patients living with chronic illnesses	• Need more coordinated care across services<br>• Cultural safety important<br>• Importance of Indigenous health workshop<br>• Service only knows Western Ways leading to cultural barrier.	QLD	Rockhamptom, Gracemere, Yeppoon
Freeman et al (59)	2014	Semi-structured interviews for staff and community assessment workshops for clients	Thematic analysis	13 Aboriginal clients<br><br>22 staff members (17 Aboriginal 5 Non-Aboriginal)	Aboriginal health service	To explore cultural respect strategies, client experiences and barriers to cultural respect.	• Clients positive about ability of service to achieve cultural respect.<br>• Clients articulated the benefits of the service through a social lens for health service provision. e.g. helping out with letters, court support documents etc.<br>• Positive impact of Aboriginal health workforce.<br>• Feeling welcome is beneficial. This comes from seeing people clients know.<br>• Transport really positive.<br>• Gender specific services important.<br>• Community culture events such as bush camps, cultural days, reconciliation events were a good healing process,<br>• Reduced trust in non-Aboriginal staff due to historical experiences	NT<br>SA	-

Gomersall et al (66)	2017	Standardised systematic review methods	Two-step thematic analysis approach of meta-aggregation	10 articles included	ACCHOs	To synthesise patient perspectives of the unique characteristics and value provided by Aboriginal Community Controlled Health Organisations (ACCHOs) compared with mainstream services.	• Appreciate outreach and transport services<br>• Familiar faces at ACCHOs<br>• Culturally safe care<br>• Taking time<br>• Provision of information that was understandable<br>• Indigenous workforce<br>• Pride in being part of local community through health service<br>• Mutual respect	-	-
Govil et al (51)	2013	Data extraction and focus groups	Thematic analysis	6 Aboriginal community members and 6 staff	AMS	To identify challenges of managing cardiovascular care in a regional AMS.	• Preference for better experience at AMS rather than MHS,<br>• Trust,<br>• Availability of more services,<br>• Confidentiality concerns as have family members working,<br>• Importance of education and family environment<br>• Extending waiting times can lead to dissatisfaction,	WA	-
Green et al (61)	2018	Semi structured in depth interviews	Thematic analysis	52 Indigenous people (17 Cancer patients, 28 health professionals, 7 both)	Cancer care services (3 public hospitals and one regional health service)	To identify key components of patients experience that should be included in patient experience measurements for Indigenous patients...	• Scare of 'dominant White medical culture'<br>• Historical trauma leading to trust concerns<br>• Indigenous health workforce more comfortable<br>• Community inclusion critical<br>• Coordination of care important<br>• Continuity develops trust<br>• Off Country concerns e.g. what would happen to spirit<br>• Importance of space e.g. flags, artwork, area for smoking ceremonies	VIC, NSW, NT	-

Harrington et al (43)	2006	Semi-structured informal interviews	Thematic analysis	15 patients 18 relatives and 18 health care workers	Primary health centre and satellite outstations	To identify reasons for failure of a RHD program by investigating factors that impact Indigenous patient compliance of medication.	• Emotional and spiritual components of care important<br>• Belonging important<br>• Hesitancy towards new health services<br>• Transportation valued<br>• Active follow up service<br>• Limited understanding of Western biomedical health<br>• Communication developing trust		
Hepworth et al (30)	2015	Open-ended interviews	Thematic analysis	7 Indigenous psychology clients, 5 social work, dietician, social worker, psychologist's and GPs, nurses, AHWs and receptionists also participated in 4 focus groups	Indigenous primary health centre	To examine the impact of integrating a mental health service in an urban Indigenous primary care service.	• Travel an issue<br>• Specifically, Indigenous services are accessible and culturally appropriate<br>• Approachable<br>• Continuity of care in same place<br>• Indigenous workforce important<br>• Some fear that Indigenous staff may know too many people in community leading to confidentiality problem	QLD	South East QLD
Homer et al (31)	2012	Focus group	Thematic analysis	7 Aboriginal mothers who had used service	Midwifery service at hospital	To explore the evaluation from Aboriginal women who had accessed a midwifery service.	• Support with access appreciated e.g. transport<br>• Continuity of care important.<br>• Trust in relationships<br>• Staff advocated for patients<br>• For some women it was first time they felt 'special' from the care given by a service	NSW	Sydney
Hughes et al (32)	2018	Discussion group at Indigenous Patient Voices Symposium 2017	Thematic analysis	24 patients	Anywhere with dialysis	To gain perspectives of kidney health care of Indigenous Australians.	• Off Country concerning<br>• Distress<br>• Isolation<br>• Cost for family to support<br>• Lack of support for family when relocating<br>• Transparency of information<br>• Biomedical model not holistic of care<br>• Communication is key	NT	-

Jan et al (62)	2004	Focus groups and interviews	Themes explored	6 focus groups of between 10-15 Aboriginal women and 35 interviews with stakeholders	AHS and hospitals	To conduct a holistic evaluation of an Aboriginal community controlled midwifery service	• Lack of relationship with hospital staff.<br>• Inadequate communication<br>• Alienation and racism<br>• Disempowering nature of hospital care for Aboriginal women. "Always thought of myself as dumb in the hospital"<br>• After the start of the Aboriginal antenatal service patients had the following experience:<br>• trust and flexibility<br>• Empowerment and family centred<br>• Ongoing relationships great<br>• Transport was positive	NSW	Western Sydney
Jobling et al (63)	2015	Focus groups and interviews	Thematic analysis	19 Aboriginal women	A range of services	To explore health service engagement and experience in urban Aboriginal women.	• Continuity of care important<br>• Rushing consultations is harmful<br>• Culturally competent healthcare important	VIC	Wurunjeri

Jowsey et al (33)	2012	Semi-structured in-depth interviews	Thematic analysis	19 Aboriginal and Torres Strait Islander people	AHS and mainstream health services	To explore how the structuring of places and time influence Indigenous patient and carer experience of health services	• AMS waiting room with strong cultural inflections positive. Meeting place set up allows it to be a social space as well.<br>• "I just, just ah come here on one of my day off and sit out here, have a talk with all my mates"<br>• Where MHSs waiting rooms are constructed as quiet and formal sick spaces, AMSs waiting rooms are constructed as meeting and speaking spaces, where people happen to be sick.<br>• In this space people can share about their illness and learn. Sharing is a cultural marker.<br>• Perceptions of time spent in the waiting room were intrinsically linked with the value that Indigenous participants placed on feeling that they had sufficient time with health professionals.<br>• Doctors take their time with each patient even if it goes over time.<br>• AMS referred to racist specialist which was negative.	ACT NSW	Western Sydney and Canberra
Kelly et al (23)	2014	Semi-structured interview	Thematic analysis	5 Aboriginal women	Continuity of care program	To explore the experience s of indigenous women who participate d in a service of having an Indigenous midwifery student.	• No feelings of shame when there is another Aboriginal person in room<br>• Makes it more comfortable and easier to understand things<br>• Trust and strength of relationship with staff important	-	-

Lowell et al (34)	2012	Semi-structured interviews	Thematic analysis	33 Yolgnu health staff, clients and other community members and non Yolgnu health staff	Local health services particularly the local primary healthcare facilities	To understand limitations in current practice related to Indigenous patients and chronic disease.	• Not given full medical background to illness is frustrating<br>• Language barrier<br>• Inadequate staff communication leading to confusion about illness<br>• Distrust in medical services	NT	Yolgnu

Mbuzi et al (70)	2017	Unstructured interviews	Thematic analysis	33 Indigenous participants including 24 patients and 9 were family members.	Hospitals	To explore the experience of hospitalisation for acute cardiac care for Indigenous Australians.	• Negative past experiences of healthcare impacting current experience<br>• Going to 'big' hospital caused concern with serious fear of dying or being very sick<br>• Staff use of difficult language<br>• Appreciated Indigenous flags and paintings but not enough as there was no Indigenous staff<br>• No one knew the language<br>• Stereotypical attitudes<br>• Disrespected when not being called aunty<br>• Elders being told what to do by younger staff<br>• Need gender separation<br>• Scared<br>• No smoking ceremonies allowed in hospitals<br>• Not comfortable sleeping on beds not cleansed of spirits of previous patients<br>• Some experiences led to patients understanding why other mob leave hospital<br>• Concerned of where hospital was built (potentially on sacred land) so had to consult with community elder before being admitted<br>• Lack of interest in Indigenous concerns if not an Indigenous staff member<br>• Would have liked to have access to a local elder in the same way religious bodies have someone<br>• Need Indigenous meeting place<br>• Felt more comfortable when meeting other Indigenous people in culturally isolating area<br>• Feeling alone when moved to different community because family can't travel due to cost	QLD	-

McGrath (77)	2006	Open-ended interviews	Thematic analysis	10 Aboriginal patients, 19 carers, 43 staff including AHWs, interpreters, and healthcare workers	Palliative care services	To explore the experience of relocation for palliative care Aboriginal patients	• Fear of relocation<br>• Scared in hospital and lonely<br>• Scared of dying off Country<br>• Fear of high tech treatments e.g. radiotherapy<br>• Fear of travel to big hospitals<br>• Language barriers	NT	Arnhem land, Elcho Island, Darwin, Alice Springs, Katherine region
McMichael et al (47)	2000	Interviews, case histories and focus group discussions	Thematic analysis	101 Indigenous women of child bearing age or older	Any/Not specified	To identify issues that affected Indigenous women with breast cancer awareness, detection, care and knowledge of support available.	• Satisfied generally<br>• Pleasant staff<br>• Radiographers explained clearly<br>• When screening occurred at Aboriginal community health centres they felt ease and safe<br>• Culturally safe<br>• Fixed appointment a barrier<br>• Appreciated explanations to be put in terms that are relatable due to sometimes a lack of understanding of terms	QLD	-
Meiklejohn et al (71)	2017	Yarning semi-structured interviews	Grounded theory, categorising	21 Indigenous people with cancer diagnosis (13 women and 8 men) (12 urban, 4 regional, 5 remote)	Hospital + primary care	To explore Indigenous cancer survivor's perspectives of follow-up cancer care and ongoing management.	• Medical jargon unhelpful<br>• Belonging at AMS<br>• Holistic care there<br>• Opportunity for social connections<br>• Transport to and from appreciated<br>• Coordination of follow up care sometimes had issues	QLD	Brisbane
Munro et al (44)	2017	In depth semi-structured interviews	Thematic analysis	21 people (12 clients and 9 staff), 17 Aboriginal people.	Drug and Alcohol Residential Rehabilitation Service	To identify perceptions of strengths and improvements areas of a remote Aboriginal drug and alcohol residential rehabilitation service.	• Embedded culture into program positive<br>• Being on country<br>• Learning culture whilst at service<br>• Allowing room for traditional spirituality<br>• Staff empathy and lived experience	NSW	Western NSW

Reilly et al (45)	2018	Face-to-face semi structured interviews	Thematic analysis	29 Aboriginal patients or cancer survivors, 11 carers, 22 service provider (8 non-Aboriginal)	Any/Not specified	To understand how care coordination impacts Aboriginal patient's experiences of cancer treatment.	• Care coordination positive<br>• Racism present and a barrier<br>• Scared about hospitals because family members die there because they don't go until really sick<br>• Gender barriers	SA	-
Rix et al (81)	2015	Semi structured interviews	Thematic analysis	18 Aboriginal patients, 29 health care providers	Renal facilities in rural NSW	To improve service delivery based on feedback of Aboriginal patients who had accessed the treatments.	• Importance of family involvement in care<br>• Strong relationship with healthcare provider<br>• Cultural safety key<br>• Previous bad experiences affecting now<br>• Fear of mainstream services	NSW	Rural
Rix et al (67)	2014	'Yarning' interviews	Thematic analysis	18 Aboriginal patients	Renal facilities in rural NSW	To describe experience and perspective of rural Aboriginal people on haemodialysis.	• Discomfort when talking about patient next to bedside<br>• Limited Western medical understanding leading to anxiety<br>• Comfortability important through time spent forming a relationship<br>• Patient's at smaller centres reported better relationships formed<br>• Medical language is a barrier	NSW	Rural
Shahid et al (80)	2011	In-depth interviews	Thematic analysis	23 Aboriginal patients, 12 survivors and 16 family members	Hospitals	To understand the experience of Indigenous patients when interacting with cancer treatment	• Transport and accommodation difficulties for rural patients and a lack of understanding of PATS<br>• No time for a cultural Welcome when they arrive on a new Country after being transported<br>• Not allowing extended family in room is difficult<br>• Intimidated and invasion of privacy with medical student groups<br>• Positive experiences when people spend time with patient and family	WA	Perth, 1 rural and 2 remote areas

Shahid et al (72)	2009	Interviews	Thematic analysis	14 Aboriginal patients and 16 family members	Hospitals	To explore Aboriginal people's views and experiences of cancer related services in WA	• Historical racism impacting treatment choices<br>• Culturally inappropriate mixed gender wards<br>• Family group mixings<br>• Gender of professionals and younger professionals providing advice for older people<br>• Language barriers<br>• Medical jargon caused frustration<br>• Lack of continuity of care means lack of strength in relationships<br>• Lack of warmth from physicians<br>• Lack of understanding of shame in private business,		Urban and regional
Shahid et al (35)	2016	In-depth open ended interviews	Thematic analysis	14 Aboriginal patients with 16 family members, 62 health professionals	Hospitals	To examine factors that contribute to delayed diagnosis of cancer among Aboriginal patients from patients and profession al's perspectiv es.	• Comments about Aboriginality negative<br>• Waiting times<br>• Mainstream not culturally safe<br>• Appreciate Aboriginal health professionals<br>• Family obligations<br>• Understanding shame	WA	Perth and rural/re mote areas
Smith et al (36)	2017	Questionnai res with qualitative questions and subscales	Thematic analysis	78 participants with 54 Aboriginal patients and others including doctors, allied staff, front desk staff, manager's board members	Primary health care services	To explore the views of stakeholde rs on cultural appropriat eness of primary health care (PHC) services for Aboriginal people.	• 46.3% of Aboriginal patients noted that communication between them and physician was clear and understandable<br>• Respectful front desk staff important<br>• Culturally appropriate space<br>• Communication<br>• Aboriginal workforce important	QLD	North West QLD

Strong et al (37)	2015	Interview	Thematic analysis	40 Aboriginal people who sought healthcare (20 men and 20 women)	Pain services	To explore communications of Aboriginal people in encounters about pain.	• Limited respect<br>• Stereotyped<br>• Embarrassed to ask for clarification<br>• Limited understanding of medical lingo<br>• Reluctance to share how they feel	QLD	South East QLD
Tam et al (73)	2018	Semi-structured interviews	Thematic analysis	12 Indigenous patients	Hospital	To explore cancer care experiences in Indigenous Australians	• Simple and clear communication<br>• Feeling valued by health professional and building relationship	QLD	-
Taylor et al (38)	2009	Open-ended interviews	Framework approach with charting of themes	12 Aboriginal patients, 11 staff	Hospital	To explore the impact that AHWs have on Aboriginal patient outcomes	• Historical impact of colonisation on current perception of hospital.<br>• "[Hospital is] the place you go to die"<br>• Patronising experiences	WA	-
Thompson et al (64)	2011	In-depth interviews	Thematic analysis	14 Aboriginal patients and 16 family members	Hospital cancer services	To understand factors that influenced Aboriginal participation in cancer care.	• Cold, indifferent and inflexible to move away from biomedical model and an absence of warm interactions<br>• Lower literacy can impact.<br>• Hospital seen as symbolising White dominance<br>• Absence of signaging such as photo and flags was noticed<br>• Family visiting hours and numbers conflicting with hospital policy<br>• Where family involvement was accepted patients appreciated it<br>• Staff turnover reflected as lack of caring as relationships over time so important<br>• Needed more culture e.g. traditional healers, medicine, smoking ceremonies<br>• Distrust and frustration<br>• Appreciated Indigenous health workforce	WA	

Treloar et al (65)	2012	Interviews	Thematic analysis	22 Aboriginal people with cancer, 18 carers, 16 health care workers	Any	Examine processes that impact on social inclusion of Aboriginal people in cancer care.	• Mistrust in institutions due to marginalisation and racism<br>• Fear of being treated differently<br>• Bad experience last time ending up discharging<br>• Did not fully understand the system	NSW	-
Webster et al (56)	2016	Focus groups	Grounded theory with coding into concepts	25 Indigenous people with diabetes (12 male and 13 female)	Any/Not specified	To explain how Aboriginal people understand and manage type 2 diabetes.	• Distrust in system<br>• Don't like messages and advice coming from non-Aboriginal people<br>• Stereotyped<br>• Positive when professionals expressed care and understanding<br>• Importance of Aboriginal health staff<br>• Communication breakdowns<br>• Dislocation from Country<br>• Family member's experience impacting them	NSW	Dubbo
Worrall-Carter et al (52)	2015	In-depth interviews	Thematic analysis	10 Aboriginal patients (6 men and 4 women)	Hospital	To better understand Aboriginal patient's experience of cardiac services.	• Dislike of hospital<br>• Unfamiliar institution<br>• Feeling dislocated on arrival due travel distance (place being an important part of Aboriginal culture)<br>• Waiting times<br>• Generally positive about engagement with staff particularly nursing staff who made an effort to bond with them<br>• Presence of AHW important<br>• Whilst generally positive a couple still experience overt racism<br>• Appreciated time taken to explain things<br>• Family critical to recovery	VIC	Melbourne and regional VIC

Wotherspoon & Williams (74)	2017	Cross sectional cohort with survey	Thematic analysis	154 participants (76 Aboriginal and/or Torres Strait Islanders and 78 comparator group)	Hospitals	To measure whether there were any in patient experience differences between Indigenous and non-Indigenous patients.	• Valued being asked Aboriginality<br>• Also valued Aboriginal health workforce<br>• AHW important<br>• Uncomfortable and scared whilst in hospital<br>• Gender issues with men treating women and vice versa<br>• Importance of family and visiting hours	VIC	Regional

References

1. Harrison R, Walton M, Manias E, Smith-Merry J, Kelly P, Iedema R, et al. The missing evidence: a systematic review of patients' experiences of adverse events in health care. International Journal for Quality in Health Care. 2015;27(6):424-42.
2. Jason A. Defining patient experience. Patient Experience Journal. 2014;1(1):7-19.
3. Feirn A, Betts D, Tribble T. The patient experience: strategies and approaches for providers to achieve and maintain a competitive advantage. United States of America: Deloitte LLP; 2009.
4. Shaw C. Patient Experience: What does it mean? : Beyong Philosophy; 2012 [Available from: https://beyondphilosophy.com/patient-experience-what-does-it-mean/.
5. Hewitson P, Skew A, Graham C, Jenkinson C, Coulter A. People with limiting long-term conditions report poorer experiences and more problems with hospital care. BMC Health Services Research. 2014;14:33.
6. Leavitt M. Medscape's response to the Institute of Medicine Report: Crossing the quality chasm: a new health system for the 21st century. Medscape General Medicine. 2001;3(2):2.
7. Donabedian A. The Quality of Care - How Can It Be Assessed. Journal of the American Medical Association. 1988;260(12):1743-8.
8. Agency for Healthcare Research and Quality. Six Domains of Health Care Quality Rockville: Agency for Healthcare Research and Quality; 2018 [Available from: https://www.ahrq.gov/talkingquality/measures/six-domains.html.
9. Doyle C, Lennox L, Bell D. A systematic review of evidence on the links between patient experience and clinical safety and effectiveness. British Medical Journal open. 2013;3(1).
10. Harrison R, Walton M, Manias E. Patients' experiences in Australian hospitals: an Evidence Check rapid review. In: Australian Commission on Safety and Quality in Health Care, editor.: Sax Institute; 2015.
11. Canadian Institute for Health Information. Patient Experience Canada: Canadian Institute for Health Information; 2019 [Available from: https://www.cihi.ca/en/patient-experience.
12. National Health System. Creating a Patient- led NHS. In: Department of Health, editor. England. 2015.
13. Australian Institute of Health and Welfare. The health and welfare of Australia's Aboriginal and Torres Strait Islander peoples. In: Australian Institute of Health and Welfare, editor. Canberra 2015.
14. Australian Commission on Safety and Quality in Health Care. National Safety and Quality Health Service Standards User Guide for Aboriginal and Torres Strait Islander Health. Sydney: Australian Commission on Safety and Quality in Health Care; 2017.
15. Kelly J, Dwyer, J., Pekarsky, B., Mackean, T., Willis, E., Battersby, M. & Glover, J. Managing Two Worlds Together: Stage 2: Patient Journey Mapping Tools. Melbourne: The Lowitja Institute; 2012.
16. Kelly J, Dwyer, J., Pekarsky, B., Mackean, T., McCabe, N., Wiseman, J., de Crespigny, C., & O'Donnell K. Managing Two Worlds Together. Stage 3: Improving Aboriginal Patient Journeys - Workbook. Melbourne: The Lowitja Institute; 2015.
17. Kelly J, Dwyer, J., Pekarsky, B., Mackean, T., Willis, E., de Crespigny, C., Perkins, S, O'Donnell,, K. K, R., Mackean, L., Brown, A., Lawrence, M. & Dixon, K. Managing Two Worlds Together. Stage 3: Improving Aboriginal Patient Journeys - Study Report. Melbourne: The Lowitja Institute; 2015.
18. Greenhalgh T, Robert G, Macfarlane F, Bate P, Kyriakidou O, Peacock R. Storylines of research in diffusion of innovation: a meta-narrative approach to systematic review. Social Science and Medicine. 2005;61(2):417-30.

19. Wong G, Greenhalgh T, Westhorp G, Buckingham J, Pawson R. RAMESES publication standards: meta-narrative reviews. BMC Medicine. 2013;11.
20. Harrison R, Walton M, Manias E, Smith-Merry J, Kelly P, Iedema R, et al. The missing evidence: a systematic review of patients' experiences of adverse events in health care. International Journal for Quality in Health Care. 2015;27(6):424-42.
21. Wolf J, Niederhauser V, Marshburn D, LaVela S. Defining Patient Experience. Patient Experience Journal. 2014;1(1):7-19.
22. Critical Appraisal Skills Programme. CASP Checklist: 10 questions to help you make sense of a Qualitative research Oxford: CASP UK; 2018 [Available from: https://casp-uk.net/wp-content/uploads/2018/03/CASP-Qualitative-Checklist-2018_fillable_form.pdf.
23. Kelly J, West R, Gamble J, Sidebotham M, Carson V, Duffy E. 'She knows how we feel': Australian Aboriginal and Torres Strait Islander childbearing women's experience of Continuity of Care with an Australian Aboriginal and Torres Strait Islander midwifery student. Women and Birth. 2014;27(3):157-62.
24. Cheng WYC, Blum P, Spain B. Barriers to effective perioperative communication in indigenous australians: an audit of progress since 1996. Anaesthesia and Intensive Care. 2004;32(4):542-7.
25. Anderson K, Cunningham J, Devitt J, Preece C, Cass A. "Looking back to my family": Indigenous Australian patients' experience of hemodialysis. BMC Nephrology. 2012;13.
26. Anderson K, Devitt J, Cunningham J, Preece C, Cass A. "All they said was my kidneys were dead": Indigenous Australian patients' understanding of their chronic kidney disease. Medical Journal of Australia. 2008;189(9):499-503.
27. Devitt J, Anderson K, Cunningham J, Preece C, Snelling P, Cass A. Difficult conversations: Australian Indigenous patients' views on kidney transplantation. BMC Nephrology. 2017;18.
28. Baba JT, Brolan CE, Hill PS. Aboriginal medical services cure more than illness: a qualitative study of how Indigenous services address the health impacts of discrimination in Brisbane communities. International Journal for Equity in Health. 2014;13.
29. Ban P. Access and attitudes to health care of Torres Strait Islanders living in mainland Australia. Australian Journal of Primary Health. 2004;10(2):29-35.
30. Hepworth J, Askew D, Foley W, Duthie D, Shuter P, Combo M, et al. How an urban Aboriginal and Torres Strait Islander primary health care service improved access to mental health care. International Journal for Equity in Health. 2015;14.
31. Homer CSE, Foureur MJ, Allende T, Pekin F, Caplice S, Catling-Paull C. 'It's more than just having a baby' women's experiences of a maternity service for Australian Aboriginal and Torres Strait Islander families. Midwifery. 2012;28(4):509-15.
32. Hughes JT, Dembski L, Kerrigan V, Majoni SW, Lawton PD, Cass A. Gathering Perspectives - Finding Solutions for Chronic and End Stage Kidney Disease. Nephrology. 2018;23:5-13.
33. Jowsey T, Yen L, Ward N, Mcnab J, Aspin C, Usherwood T, et al. It hinges on the door: Time, spaces and identity in Australian Aboriginal Health Services. Health Sociology Review. 2012;21(2):196-207.
34. Lowell A, Maypilama E, Yikaniwuy S, Rrapa E, Williams R, Dunn S. "Hiding the story": Indigenous consumer concerns about communication related to chronic disease in one remote region of Australia. International Journal of Speech-Language Pathology. 2012;14(3):200-8.
35. Shahid S, Teng THK, Bessarab D, Aoun S, Baxi S, Thompson SC. Factors contributing to delayed diagnosis of cancer among Aboriginal people in Australia: a qualitative study. British Medical Journal Open. 2016;6(6).
36. Smith K, Fatima Y, Knight S. Are primary healthcare services culturally appropriate for Aboriginal people? Findings from a remote community. Australian Journal of Primary Health. 2017;23(3):236-42.

37. Strong J, Nielsen M, Williams M, Huggins J, Sussex R. Quiet about pain: Experiences of Aboriginal people in two rural communities. Australian Journal of Rural Health. 2015;23(3):181-4.
38. Taylor KP, Thompson SC, Smith JS, Dimer L, Ali M, Wood MM. Exploring the impact of an Aboriginal Health Worker on hospitalised Aboriginal experiences: lessons from cardiology. Australian Health Review. 2009;33(4):549-57.
39. Brown A. Acute Coronary Syndromes in Indigenous Australians: Opportunities for Improving Outcomes Across the Continuum of Care. Heart, Lung and Circulation. 2010;19(5-6):325-36.
40. Campbell S, Brown S. Maternity care with the Women's Business Service at the Mildura Aboriginal Health Service. Australian and New Zealand Journal of Public Health. 2004;28(4):376-82.
41. Corcoran PM, Catling C, Homer CSE. Models of midwifery care for Indigenous women and babies: A meta-synthesis. Women and Birth. 2017;30(1):77-86.
42. Einsiedel LJ, van Iersel E, Macnamara R, Spelman T, Heffernan M, Bray L, et al. Self-discharge by adult Aboriginal patients at Alice Springs Hospital, Central Australia: insights from a prospective cohort study. Australian Health Review. 2013;37(2):239-45.
43. Harrington Z, Thomas DP, Currie BJ, Bulkanhawuy J. Challenging perceptions of non-compliance with rheumatic fever prophylaxis in a remote Aboriginal community. Medical Journal of Australia. 2006;184(10):514-7.
44. Munro A, Allan J, Shakeshaft A, Breen C. "I just feel comfortable out here, there's something about the place": staff and client perceptions of a remote Australian Aboriginal drug and alcohol rehabilitation service. Substance Abuse Treatment, Prevention, and Policy. 2017;12.
45. Reilly R, Micklem J, Yerrell P, Banham D, Morey K, Stajic J, et al. Aboriginal experiences of cancer and care coordination: Lessons from the Cancer Data and Aboriginal Disparities (CanDAD) narratives. Health Expectations. 2018;21(5):927-36.
46. Fredericks B, Kinnear S, Daniels C. Exploring knowledge, experiences and perceptions of chronic health conditions. Aboriginal and Islander Health Worker Journal. 2016;40:17-8.
47. McMichael C, Kirk M, Manderson L, Hoban E, Potts H. Indigenous women's perceptions of breast cancer diagnosis and treatment in Queensland. Australia and New Zealand Journal of Public Health. 2000;24(5):515-9.
48. Canuto K, Wittert G, Harfield S, Brown A. "I feel more comfortable speaking to a male": Aboriginal and Torres Strait Islander men's discourse on utilizing primary health care services. International Journal for Equity in Health. 2018;17(185).
49. Artuso S, Cargo M, Brown A, Daniel M. Factors influencing health care utilisation among Aboriginal cardiac patients in central Australia: a qualitative study. BMC Health Services Research. 2013;13.
50. Burnette L, Kickett M. 'You are just a puppet': Australian Aboriginal people's experience of disempowerment when undergoing treatment for end-stage renal disease. Renal Society of Australasia Journal. 2009;5(3):113-8.
51. Govil D, Lin I, Dodd T, Cox R, Moss P, Thompson S, et al. Identifying culturally appropriate strategies for coronary heart disease secondary prevention in a regional Aboriginal Medical Service. Australian Journal of Primary Health. 2014;20(3):266-72.
52. Worrall-Carter L, Daws K, Rahman MA, MacLean S, Rowley K, Andrews S, et al. Exploring Aboriginal patients' experiences of cardiac care at a major metropolitan hospital in Melbourne. Australian Health Review. 2016;40(6):696-704.
53. Kuhn TS. The Structure of Scientific Revolutions. 2d ed. Chicago,: University of Chicago Press; 1970. xii, 210 p. p.
54. Chapman R, Smith T, Martin C. Qualitative exploration of the perceived barriers and enablers to Aboriginal and Torres Strait Islander people accessing healthcare through one Victorian Emergency Department. Contemporary Nurse. 2014;48(1):48-58.

55. Foley W, Houston A. Closing the gap by increasing access to clinical dietetic services for urban Aboriginal and Torres Strait Islander people. Nutrition & Dietetics. 2014;71(4):216-22.
56. Webster E, Johnson C, Kemp B, Smith V, Johnson M, Townsend B. Theory that explains an Aboriginal perspective of learning to understand and manage diabetes. Australian and New Zealand Journal of Public Health. 2017;41(1):27-31.
57. Fredericks B. Utilising the Concept of Pathway as a Framework for Indigenous Research. Australian Journal of Indigenous Education. 2007;36:15-22.
58. Aspin C, Brown N, Jowsey T, Yen L, Leeder S. Strategic approaches to enhanced health service delivery for Aboriginal and Torres Strait Islander people with chronic illness: a qualitative study. BMC Health Services Research. 2012;12.
59. Freeman T, Edwards T, Baum F, Lawless A, Jolley G, Javanparast S, et al. Cultural respect strategies in Australian Aboriginal primary health care services: beyond education and training of practitioners. Australian and New Zealand Journal of Public Health. 2014;38(4):355-61.
60. Brener L, Wilson H, Jackson LC, Johnson P, Saunders V, Treloar C. Experiences of diagnosis, care and treatment among Aboriginal people living with hepatitis C. Australian and New Zealand Journal of Public Health. 2016;40:59-64.
61. Green M, Anderson K, Griffiths K, Garvey G, Cunningham J. Understanding Indigenous Australians' experiences of cancer care: stakeholders' views on what to measure and how to measure it. BMC Health Services Research. 2018;18(1):982.
62. Jan S, Conaty S, Hecker R, Bartlett M, Delaney S, Capon T. An holistic economic evaluation of an Aboriginal community-controlled midwifery programme in Western Sydney. Journal Health Services Research Policy. 2004;9(1):14-21.
63. Jobling K, Lau P, Kerr D, Higgins RO, Worcester MU, Angus L, et al. Bundap Marram Durn Durn: Engagement with Aboriginal women experiencing comorbid chronic physical and mental health conditions. Australian and New Zealand Journal of Public Health. 2016;40:30-5.
64. Thompson SC, Shahid S, Bessarab D, Durey A, Davidson PM. Not just bricks and mortar: planning hospital cancer services for Aboriginal people. BMC Research Notes. 2011;4:62.
65. Treloar C, Gray R, Brener L, Jackson C, Saunders V, Johnson P, et al. "I can't do this, it's too much": building social inclusion in cancer diagnosis and treatment experiences of Aboriginal people, their carers and health workers. International Journal of Public Health. 2014;59(2):373-9.
66. Gomersall JS, Gibson O, Dwyer J, O'Donnell K, Stephenson M, Carter D, et al. What Indigenous Australian clients value about primary health care: a systematic review of qualitative evidence. Australian and New Zealand Journal of Public Health. 2017;41(4):417-23.
67. Rix EF, Barclay L, Stirling J, Tong A, Wilson S. 'Beats the alternative but it messes up your life': Aboriginal people's experience of haemodialysis in rural Australia. British Medical Journal Open. 2014;4(9).
68. Conway J, Lawn S, Crail S, McDonald S. Indigenous patient experiences of returning to country: a qualitative evaluation on the Country Health SA Dialysis bus. BMC Health Services Research. 2018;18.
69. Davies J, Bukulatjpi S, Sharma S, Davis J, Johnston V. "Only your blood can tell the story" - a qualitative research study using semi-structured interviews to explore the hepatitis B related knowledge, perceptions and experiences of remote dwelling Indigenous Australians and their health care providers in northern Australia. BMC Public Health. 2014;14.
70. Mbuzi V, Fulbrook P, Jessup M. Indigenous cardiac patients' and relatives' experiences of hospitalisation: A narrative inquiry. Journal of Clinical Nursing. 2017;26(23-24):5052-64.

71. Meiklejohn JA, Garvey G, Bailie R, Walpole E, Adams J, Williamson D, et al. Follow-up cancer care: perspectives of Aboriginal and Torres Strait Islander cancer survivors. Supportive Care in Cancer. 2017;25(5):1597-605.
72. Shahid S, Finn L, Bessarab D, Thompson SC. Understanding, beliefs and perspectives of Aboriginal people in Western Australia about cancer and its impact on access to cancer services. BMC Health Services Research. 2009;9.
73. Tam L, Garvey G, Meiklejohn J, Martin J, Adams J, Walpole E, et al. Exploring Positive Survivorship Experiences of Indigenous Australian Cancer Patients. International Journal of Environmental Research and Public Health. 2018;15(1).
74. Wotherspoon C, Williams CM. Exploring the experiences of Aboriginal and Torres Strait Islander patients admitted to a metropolitan health service. Australian Health Review. 2019;43(2):217-23.
75. Harrison R, Walton M, Manias E, Mears S, Plumb J. Patients' experiences in Australian hospitals: a systematic review of evidence. Australian Health Review. 2017;41(4):419-35.
76. Walker M, Fredericks B, Mills K, Anderson D. "Yarning" as a Method for Community-Based Health Research With Indigenous Women: The Indigenous Women's Wellness Research Program. Health Care for Women International. 2014;35(10):1216-26.
77. McGrath P. Exploring Aboriginal peoples' experience of relocation for treatment during end-of-life care. International Journal of Palliative Nursing. 2006;12(3):102-8.
78. National Aboriginal Health Strategy Working Party. National Aboriginal Health Strategy. Canberra: Commonwealth of Australia; 1989.
79. Dembinsky M. Exploring Yamatji perceptions and use of palliative care: an ethnographic study. International Journal of Palliative Nursing. 2014;20(8):387-93.
80. Shahid S, Finn L, Bessarab D, Thompson SC. 'Nowhere to room ... nobody told them': logistical and cultural impediments to Aboriginal peoples' participation in cancer treatment. Australian Health Review. 2011;35(2):235-41.
81. Rix EF, Barclay L, Stirling J, Tong A, Wilson S. The perspectives of Aboriginal patients and their health care providers on improving the quality of hemodialysis services: A qualitative study. Hemodialysis International. 2015;19(1):80-9.
82. Dwyer J, Kelly, J., Willis, E., Glover, J., Mackean, T., Pekarsky, B. & Battersby, M. . Managing Two Worlds Together: City Hospital Care for Country Aboriginal People - Project Report. Melbourne: The Lowitja Institute; 2011.
83. Bond C, Brough M, Willis J, Stajic J, Mukandi B, Canuto C, et al. Moving beyond the front line: A 20-year retrospec ve cohort study of career trajectories from the Indigenous Health Program at the University of Queensland. Brisbane: University of Queensland; 2018.
84. Herring S, Spangaro J, Lauw M, McNamara L. The Intersection of Trauma, Racism, and Cultural Competence in Effective Work with Aboriginal People: Waiting for Trust. Australian Social Work. 2013;66(1):104-17.
85. Krieg A. The experience of collective trauma in Australian Indigenous communities. Australasian Psychiatry: Bulletin of Royal Australian and New Zealand College of Psychiatrists. 2009;17 Suppl 1:S28-32.
86. Muskett C. Trauma-informed care in inpatient mental health settings: a review of the literature. International Journal of Mental Health Nursing. 2014;23(1):51-9.
